# The experiences of podiatrists prescribing custom foot orthoses and patients using custom foot orthoses for foot pain management in the United Kingdom: A focus group study

**DOI:** 10.1002/jfa2.12047

**Published:** 2024-08-21

**Authors:** Emily Leach, Emma Cowley, Catherine Bowen

**Affiliations:** ^1^ School of Health Sciences Faculty of Environmental and Life Sciences University of Southampton Southampton UK; ^2^ Solent NHS Trust Podiatry Solent NHS Trust, Southampton Southampton UK; ^3^ Centre for Sport Exercise and Osteoarthritis Versus Arthritis University of Southampton Southampton UK

**Keywords:** custom foot orthoses, foot orthoses, foot pain, patient experience, podiatrist experience

## Abstract

**Introduction:**

Foot pain can be a significant burden for patients. Custom foot orthoses (CFOs) have been a mainstay in podiatry treatment for foot pain management and improving foot function. However, little is known about podiatrists' experience of prescribing CFOs or patient experience of using foot orthoses (FOs), including CFOs, for foot pain.

**Methods:**

A focus group (FG) discussion with three FOs users (Female = 2 and Male = 1) was conducted in November 2022 within a private podiatry practice. This group represented non‐experts from the general local population of individuals with existing or previous foot pain who have personally experienced using either over‐the‐counter FOs or CFOs. An online FG discussion with five musculoskeletal (MSK) specialist podiatrists (Female = 2 and Male = 3) was also conducted in December 2022. This group represented podiatrists with specialist knowledge in foot biomechanics and clinical experience in CFO provision. The FG discussions were recorded and lasted 49 and 57 min respectively. Transcribed data was manually coded, and a thematic analysis was undertaken to identify patterns within the collected data.

**Results:**

The participants in the patient FG detailed mixed experiences of the prescription process and CFOs received, with reports of limited involvement/input in their prescription, the need for frequent adjustments and high costs. The impact on footwear choices, replicability and transferability of CFOs into different types of shoes and technologies to aid design were also highlighted. In the podiatrist FG, lack of confidence in design and manufacture processes, prescription form language, relationship and communication building with manufacturers, variability in the CFOs issued and the need for better student education in CFO provision emerged as key themes.

**Conclusion:**

Patients and podiatrists shared similar views on CFO provision, namely poor communication with manufacturers leading to dissatisfaction with the CFOs prescribed causing negative impacts on patient experiences. Podiatrists called for greater education at registration level to increase new graduate podiatrist knowledge in CFO design and manufacture and better collaboration with manufacturing companies.

**Abbreviations**
3D: three‐dimension
CAD/CAM: computer aided design/computer aided manufacturing
CFOs: custom foot orthoses
FOs: foot orthoses
HCPC: Health and Care Professions Council
MSK: musculoskeletal
NHS: National Health Service
UK: United Kingdom

## 1 INTRODUCTION

Within primary care in the United Kingdom (UK), there are high consultation rates for foot and ankle musculoskeletal (MSK) problems [1, 2], with estimates showing that between 15% and 36% of people report foot complications [3]. Foot pain has been identified as an independent risk factor for lessened resilience to chronic long‐term conditions, loss of independence and reduced quality of life [4, 5]. Therefore, optimising foot health is vital for all aspects of wellbeing, participation in activity and general health [6].

The UK foot health industry comprises approximately 13,000 podiatrists trained in orthosis provision [7]. Custom foot orthoses (CFOs) are used as a conservative therapeutic treatment option to improve foot and lower limb function and offload painful structures during weight‐bearing activities and to reduce pain [8]. 2017 survey data reported that almost half of UK podiatrists issue 11–50 pairs of foot orthoses (FOs), including CFOs, a month, increasing to 100 pairs a month for 14% [9].

There are large variations in CFO prescription [10]. Comprehensive development of guidelines for the prescription of CFOs for foot pathologies is required [11]. CFOs typically require clinicians to perform both an objective and subjective assessment to arrive at a working diagnosis where a prescription is then formulated to the patient's requirements. The biomechanical theories that underpin objective foot assessments have changed over time [12] and subjective assessments are reliant upon clinician experience and preference, making the link between assessment findings and CFO prescription variables increasingly challenging. The clinician captures the morphology of the patient's foot or feet using one of a number of methods including plaster casts, foam impression boxes or three‐dimensional (3D) scanning [13]. In the past decade, there has been a move towards sending the 3D scan files or cast/foam impressions alongside the patient's prescription to the manufacturer where adjustments are made based on geometry and clinician specifications [14, 15].

The effectiveness of CFOs is variable [16] and this may be due to design and prescription processes that are non‐specific. There is good evidence for the efficacy of CFOs to manage foot conditions including diabetes and rheumatoid arthritis [9, 17]. However, evidence supporting CFOs as a treatment option for foot pathologies, such as pes planus, is fairly inconclusive [11]. Poor patient experience, low levels of satisfaction, increased pain and the need for frequent adjustments have been shown to affect the efficacy of FO therapy and patient satisfaction [18].

Foot pain is a considerable healthcare burden, yet better access to CFO provision across the UK is needed and prescription processes for optimal management of foot pain require refinement to ensure positive patient experiences. This study aimed to investigate experiences of CFO design and modifications, levels of satisfaction, thoughts on the overall prescription process and the effect of CFOs on foot pain through patient and podiatrist perspectives.

## 2 MATERIALS AND METHODS

### 2.1 Study design

As this study aimed to gather the thoughts and experiences of podiatrists prescribing CFOs and patient experiences of using CFOs, a qualitative approach was adopted. The qualitative research method selected for this study used two focus groups (FGs) as there was a need for interaction and group engagement to inform the best possible feedback [19].

### 2.2 Study recruitment

The study attempted to recruit five patients who have used FOs, including CFOs, for foot pain management and five podiatrists with experience prescribing CFOs through convenience sampling. As this study was explorative, a small sample is deemed sufficient [20]. Convenience sampling was chosen as it allows the researcher to recruit participants according to their availability and accessibility [21]. Recruitment rate to the patient FG was low. The authors propose that this was due to limited recruitment venues, strict inclusion criteria (Table 1) and the requirement for participants to express their views. In total, three participants were recruited to the patient FG and five participants were recruited to the podiatrist FG.

**TABLE 1:** Eligibility criteria for patient focus group.

Inclusion criteria	Exclusion criteria
Previous or current foot pain.	Unable to participate or communicate in a semi structured focus group.
Has experienced podiatry services.	Unable to cognitively participate in a focus group.
Has experienced personal use of FOs for any length of time at any stage of life.	Under 18 years old.
Have access to email facilities for correspondence.	Personal financial affiliations with industries with a conflict of interest to FOs manufacturing services.
	Financial investment with or involvement (such as directorships) in any organisations or entity with any financial interest in 3D print manufacture, equipment or systems.

The patient sample was recruited from a single private podiatry practice local to Southampton. This setting was chosen because patients using this service have foot pain and access to FOs. The podiatrist sample was recruited through the University of Southampton podiatry clinical educator expert reference group network. Participants were assessed on their suitability by inclusion/exclusion criteria (Table 1 and Table 2), and if eligible for the study, they were provided with contact information for the researcher [E.L.].

**TABLE 2:** Eligibility criteria for podiatrist focus group.

Inclusion criteria	Exclusion criteria
Health and Care Professions Council (HCPC) UK registered podiatrist.	Unable to participate or communicate in a semi‐structured focus group.
Five years' experience as a podiatrist with experience in MSK clinics AND more than 1 years' experience as a MSK podiatrist prescribing CFOs.	Unable to cognitively participate in a focus group.
Weekly clinics which include treating adults with MSK lower limb injuries.	Under 18 years old.
Have access to email facilities for correspondence.	Personal financial affiliations with industries with a conflict of interest to FOs manufacturing services.
	Financial investment with or involvement (such as directorships) in any organisations or entity with any financial interest in 3D print manufacture, equipment or systems.

Participants were fully informed of the protocol. Written informed consent was gathered to make sure they were participating voluntarily. The University of Southampton Ethics and Research Governance Online (ERGO) system approved the study (ERGO 72500).

## 3 DATA COLLECTION

The patient FG was held in November 2022 at a local private practice with the seating laid out in a ‘banquet style’ arrangement to maximise participation and engagement. The podiatrist FG was held online via Microsoft Teams in December 2022. The patient FG lasted 49 min and the podiatrist focus group lasted 57 min. Both focus groups were convened by researchers [E.C.] and [E.L.]. The FG schedules were developed by [E.C.] with input from [E.L.] (Supporting Information S1). Participants were encouraged to speak broadly, and researchers used prompts to probe for relevant information and to follow‐up on any emerging themes [22]. The patient FG was audio‐recorded using an Olympus digital recorder and transcribed verbatim (Supporting Information S1). The podiatrist FG was audio‐recorded and transcribed using the audio recording and transcription capabilities within Microsoft Teams (Supporting Information S1).

## 4 DATA ANALYSIS

Thematic analysis was employed to analyse the transcribed data. Braun and Clarke's (2006) six‐phase guide [23] (Supporting Information S1) was adopted as it is a reliable framework for thematic analysis and has been used extensively across a multitude of disciplines, many of which often include a health focus [24, 25].

After a period of familiarisation through immersion in the data by reading and reviewing the FG transcripts, an initial coding scheme (Supporting Information S1) was developed by [E.L.] who has previous experience in thematic analysis. Exemplars from the dialogue were extracted to demonstrate truthfulness of the data within each theme.

## 5 RESULTS

The characteristics of the three participants in the patient focus group are detailed in Table 3. The characteristics of the five participants in the podiatrist focus group are detailed in Table 4.

**TABLE 3:** Participant characteristics for the patient focus group.

Participant id	Sex	Age	FO provision	Other therapies	Foot pain
1	Female	Elderly	CFO	Yes–exercise rehabilitation	Ongoing
2	Male	Middle‐aged	CFO	Yes–exercise rehabilitation	Ongoing
3	Female	Middle‐aged	Over‐the‐counter	No	Resolved

**TABLE 4:** Participant characteristics for the podiatrist focus group.

Participant id	Sex	Current region of practice	Private or National Health Service (NHS)
1	Male	South	Both
2	Male	South	Private
3	Female	South West	NHS
4	Male	South West	Both
5	Female	South West	NHS

In terms of patient and podiatrist experiences, the themes that emerged were communication, relationships and collaboration. The themes associated with process that emerged through the patient and podiatrist FG discussions were translation and transferability. Technologies also emerged as a key theme from both discussions (Table 5).

**TABLE 5:** Key themes from focus group discussions.

Key themes	
Experience	Communication, relationships and collaboration
Process	Translation and transferability
Technologies	Environment, access, 3D printing and computer‐aided design/computer‐aided manufacture (CAD/CAM)

### 5.1 Experiences

Participants in the patient FG felt it was important that they were listened to and involved in decisions regarding their foot pain management. They felt consultation times needed to be longer as the process felt rushed, and they needed more time to talk with the podiatrist.

P2: “It’s important to have that voice and give that feedback because things change all the time.”

P2: “I think to have more time to talk [would be good]. It is quite focussed. You’re in there, you get measured, get stuff next time and then you’re done, it’s quite clinical and targeted… some discussion like what’s your experience? What’s it like when walking and standing?”

Podiatrist FG participants agreed the current language used in prescription forms was not transferrable between the podiatrist and manufacturer and establishing a good relationship with manufactures helped to reduce the number of modifications needed.

P2: “All the language is different… everyone's 5° is slightly different… Sometimes I don't even use their lab form. I write it in my own language, and they know what I mean which is OK for me, but that's not scalable at all for a profession.”

P3: “You get variations amongst clinicians… what I ask for and what my colleagues ask for although we think we're asking for the same thing, if we use slightly different terminology, you'll get different things back.”

P2: “It's imperative that you build up trust in the lab so that you have confidence or at least more confidence in them…the number of modifications we have to do are very minimal because I think it goes back to building that relationship with the lab.”

### 5.2 Process

Patient FG participants discussed their FOs journeys with reports of the process being long, often having to see numerous podiatrists and many iterations/adjustments of their CFOs. They discussed ways of improving the current process.

P1: “I went to see several podiatrists in XXX and they all have their different ways of doing things. Standing on something or using a digital device whilst lying on a couch but I couldn’t see how good that was.”

P2: “Maybe there is a better solution like contact lenses instead of glasses… you have your eyes retested each year and I think it is even more important with your feet as they are a more important part of the body. What you need in an orthotic can be changed overtime and change even more so than your sight or teeth.”

Limitations of only having one pair of CFOs and having to transfer them between different footwear was discussed with patient FG participants agreeing multiple pairs of CFOs would be useful especially for different types of footwear, events and exercise.

P2: “For me, the difficulty is you only have one set, so you have to keep taking them out. I went for a run on Tuesday and it rained so they got so wet so you have to walk around in them either in wet shoes or don’t have them and your feet get sore so what do you do? It would be good to have an efficient way to have more than one pair.”

P2: “Ultimately, I want to scan the thing [foot] at home and have lots of pairs [of CFOs] for walking, work and sport.”

The process of completing a prescription form, the usefulness of these forms and how these forms are translated by manufacturers was discussed during the podiatrist FG.

P1: “Despite the loads and loads of orthotics labs and lots of work that's been done in trying to design the perfect prescription form, I don't think that exists… they're too complicated.”

P5: “The forms are quite good because they do try and make you think and they kind of give you a tick list to go.”

P1: “Some of the casts that arrived [to the manufacturer], they don't even look like they've been taken from a foot… they’re [the manufacturer is] like, how on Earth am I supposed to [interpret that] and try to design it by reading the podiatrist’s [prescription].”

### 5.3 Technologies

The differences between prescription of CFOs and available technologies in UK podiatry private practice versus National Health Service (NHS) podiatry services were compared during the podiatrist FG.

P3: “We've changed labs say three times and that has been out of our control because the way [XXX NHS Trust] works.”

P5: “I work in a different part of [XXX NHS Trust] to [P3] and we don't have access to the same things.”

Participants 1 and 2, who work in private practice, discussed the benefits of 3D scanning and CAD/CAM technologies in detail.

P1: “I got involved in CAD/CAM technology about eight years ago… I design the orthotic myself. It doesn't go to the lab… you can shape it, you can cut out, you look at it and go now that's what I want. There's no argument about whose fault it was if it didn't work because I designed it.”

P2: “The digital scanning I've been doing it for six years now and that makes my life a lot easier.”

Participant 4 looked towards the future with a positive outlook on the opportunities for expansion of these technologies within podiatry and the benefit to patients.

P4: “I think [3D printing] would give the best patient outcome in terms of consistency of device, quality of device and then also there's return being so much quicker.”

P4: “Patients like that experience as well because they think it's new technology… it just adds a bit more theatre to that patient experience and then they come back and they see their foot and they're like oh wow, that's my foot…. It all becomes that nice journey for the patient.”

This is supported by expressions voiced during the patient FG.

P2: “I want to see the mechanics…more technological, I’d quite like that.”

The podiatrist FG participants considered the usefulness of embedding teaching in CFO design technologies within BSc Podiatry programmes.

P1: “I'm not seeing a lot of podiatrists that are sitting down doing CAD/CAM. if I could change something, I would say teach it in University.”

P4: “CAD/CAM design at University would be really valuable… much greater depth of understanding of how these devices are made and how the device might return and what they might be thinking about with their prescriptions at that level.”

## 6 DISCUSSION

This study provides insight into the experiences of users and prescribers of CFOs. Our results highlight variation in expectations of the prescription and application processes for CFOs between podiatrists and patients. Podiatrists in our study concentrated more on the scanning and design processes of CFOs, whereas patients focused more on the effect of the device on their foot pain. The participants in the patient FG highlighted the need for shared decision‐making and the need for multiple sets of CFOs to be prescribed. Patients and podiatrists agreed that the current process is suboptimal. Podiatrists highlighted that the process requires refinement in terms of application of technology, prescription form language and education at the beginning of podiatry careers.

### 6.1 Experience

Previous work on user perspectives on outcomes for orthotic interventions reported patients having dissatisfaction with their current orthotic allowance and identified patient–clinician relationship as an important aspect of care [26]. The patients involved in this study raised similar points emphasising the need to be involved in decisions regarding their foot pain management. Shared decision‐making is advocated by the National Institute for Health and Care Excellence (2021) guidelines [27] [NG197] and is a key component of universal personalised care [28]. Research has shown how a good patient–clinician relationship can increase the likelihood of adherence with medical interventions [29], patient satisfaction [30] and maintain continuity in care [31]. The facilitation of shared responsibility is important for those with long‐term conditions, such as foot pain, and so is relevant to MSK podiatry service delivery [32]. Health Education England (2021) has developed a roadmap which actively takes a personalised care approach to enable shared decision‐making which should be adopted [33]. In terms of CFO provision, there is little research on the effect of shared decision‐making on patient outcomes. This is surprising given the consequent number of CFOs prescribed and the costs to the health service if CFOs are not fit for purpose.

Patient FG participants had generally positive opinions of the podiatrists involved in their care; however, one participant noted that their consultation seemed rushed. We are unable to provide comment on podiatrists' thoughts on consultation times as themes surrounding this did not emerge during the FG discussion. However, we appreciate that clinicians may face constraints, such as short consultation times, which may limit their ability to communicate fully to the patient.

Participants in the patient FG had mixed experiences in terms of levels of satisfaction of their FOs when considering comfort and pain. There is good evidence for the use of CFOs for certain conditions linked to foot pain, including diabetes [34] and rheumatoid arthritis [35], in terms of reducing risks of ulceration and plantar pressures. Patient experiences and comfort evaluation are often overlooked when evaluating the effectiveness of FOs in the literature. More effort should be made to address these factors in future CFO research given that the perception of poor comfort may lead to poor user compliance [36].

### 6.2 Process

The podiatrists within this study explored the theme of communication in great depth. Poor communication in healthcare is associated with various negative outcomes: discontinuity of care, compromise of patient safety, patient dissatisfaction and inefficient use of valuable resources [37]. In CFO provision, there are opportunities for breakdown in communication between patients, podiatrists and manufacturers given the differing knowledge sets and terminologies used between professions. Some patients in this study had a poor understanding of the intricacies of CFO prescription and design processes. P1 could not comprehend how useful a non‐weight‐bearing 3D scan of the foot could be despite the literature highlighting the quality of the 3D scan has a profound impact on the final device [38, 39]. Patient engagement and understanding is essential to the effective delivery of care [40], therefore ensuring patients are fully informed of the rationale behind assessment choices and techniques for taking foot impressions will be important for improving patient understanding of CFO prescription and design processes. Collaboration between professions has been shown to positively improve patient outcomes [41]. In CFO provision, collaboration of podiatrists and manufacturers on a common goal to refine design and prescription processes to reduce the need for further modifications and reduce the burden of visits on patients and the healthcare system needs to be a priority [42].

Provision of CFOs has the potential to achieve significant health, quality of life and economic benefits for people with foot pain and health service commissions [43]. Access to NHS podiatry services for foot pain is challenging. Currently, the majority of people with foot pain are referred to orthopaedics and physiotherapy, with fewer than half being referred to podiatry [44] and many NHS podiatry services are being predominantly funded to treat people who have diabetes and foot ulceration [45]. It is difficult to estimate the number of people issued with CFOs by podiatrists given that there are complexities in the pathways of care and differences in availability and accessibility across the country. The participants in the podiatrist FG highlighted the impact of NHS podiatry services reconfiguration of skill mix and services away from management of foot pain to focus on foot ulcer management, which may mean that more people are seeking private podiatry for foot pain management as there are more assessment and treatment options available to patients.

### 6.3 Technologies

Advancing technologies in healthcare have been shown to have positive effects on patient experience [46]. Participants highlighted design technologies, such as CAD/CAM, 3D scanning and 3D printing has the potential to revolutionise MSK podiatry practice. 3D printing and CAD/CAM have been shown to improve patient outcomes, streamline processes thereby lowering costs, increasing quality of the end product and enhancing the efficiency of orthoses delivery [47, 48]. CAD/CAM technologies are growing in popularity across the field of prosthetics and orthotics as although 3D scanning and CAD/CAM methods of manufacture require the same clinician experience and skill as hands on methods; this gives the prosthetist/orthotist full control over the design whilst preserving a digital record of individual designs allowing for easier design replicability and lower margins of error [49]. However, until the use of these technologies becomes common place in podiatry practice, it is hard to comment on and compare this method of CFO manufacture compared to current processes using external manufacturers.

Participants in the podiatrist FG highlighted the importance of professional development in the design and manufacturing of CFOs. It may be inferred that newer graduates may be keener to embrace emerging technologies, while older graduates may not. However, the information gathered through this study is unable to comment on this. Nevertheless, as understanding the key concepts of the knowledge base relevant to their profession (which includes provision of CFOs for foot pain management and prevention) is a core competency within the Health and Care Professions Council (HCPC) standards of proficiency for podiatrists, it is important that CFOs design and manufacture and associated technologies involved in this are being taught at graduate level and consolidated through clinical practice placement as identified in the HCPC standards stated in the curriculum [50].

### 6.4 Strengths and limitations

Through facilitated discussion, participants were able to build on each other's ideas. Taking a semi‐structured approach to FG discussion allowed for greater freedom for participants to express their views and more in‐depth information was gathered. The FG was facilitated by a member of the research team [C.B.] with prior experience in conducting FGs, and the transcripts were coded and thematically analysed by a member of the research team [E.L.] with prior training in this area.

Despite the participants involved in this study being representable to the CFO user and provider populations, the study was subject to bias. Firstly, inclusive bias through convenience sampling may have occurred affecting the transferability of results. It is unlikely that data saturation was reached through conducting a single patient and podiatrist FG; new ideas or themes may have been identified in later FGs. Secondly, if more participants were recruited to the patient FG, then there would be greater depth of discussion and more opportunities to follow‐up on questions and themes. Additionally, as two of the participants in the podiatrist FG had an existing relationship with at least one of the researchers, the study is subject to social desirability bias. Participants may also have answered questions untruthfully and aimed to please the researchers instead of providing unfavourable opinions [51]. FGs have the potential to encounter interviewer bias as the interviewers may subconsciously give subtle clues that influence the participant into giving answers skewed towards the interviewer's own opinions [52]. It would have been useful to employ a neutral researcher to conduct the FGs to avoid this. However, this was not possible given the time and resources available to the research team. Thirdly, we acknowledge that the study findings are based on the views of the patients and podiatrists interviewed during the FGs, and the study is subject to bias, so the results must be interpreted in the light of this. In this study, we specifically recruited patients using FOs for foot pain. Experiences of some patients who may use FOs for other reasons, such as managing foot pathology or for offloading purposes, are not included in this investigation and may differ. Similarly, some types of foot pain have an underlying cause that may not be able to be resolved through the use of FOs and may have reported different experiences. Experiences of our participants are also limited in that our recruitment to the patient FG was through a single private practice and not a UK NHS site. The experiences of the patients involved in this study may not be generalisable to FOs users through the NHS; however, they are representative of FOs users through the private sector, which arguably represents the higher user population in the UK given the changing landscape of MSK practice in NHS podiatry services [45].

## 7 CONCLUSION

Patient experiences with MSK podiatry services for FO provision, including CFOs, were fairly mixed. All of the patients reported a reduction in pain since using FOs; however, patients were frustrated with a lack of involvement in their care and inability to receive multiple pairs of CFOs. The podiatrists had clear thoughts on the limitations within the current CFO design and prescription process and emphasised the need for clearer prescription forms, more streamlined and coherent communication with manufacturers and training of newly qualified staff focused on CFO design and prescription and engagement with new technologies. The ultimate aim of CFO design and prescription is to improve patient foot pain. Through refining design and prescription processes, this will reduce the number of modifications required which will save clinician and patient time and costs which will improve the fit of the CFOs and the intervention outcome.

## AUTHOR CONTRIBUTIONS

**Emily Leach**: Conceptualization; methodology; investigation; writing – original draft; writing – review & editing. **Emma Cowley**: Conceptualization; methodology; investigation; writing – review & editing; supervision. **Catherine Bowen**: Conceptualization; methodology; investigation; writing – review & editing; supervision.

## CONFLICT OF INTEREST STATEMENT

Catherine Bowen is an emeritus editor of the Journal of Foot and Ankle Research. All other authors on this paper declare that they have no conflicts of interest.

## ETHICS STATEMENT

The faculty of Health Sciences at the University of Southampton approved the study (ERGO 72500) and all participants signed written consent.

## PATIENT CONSENT STATEMENT

Participants consent to publish was granted.

## Supporting information

Supporting Information S1

## Data Availability

The authors confirm that the data supporting the findings of this study are available within the article and its supporting information.
