# Supplementary material for: The experiences of podiatrists prescribing custom foot orthoses and patients using custom foot orthoses for foot pain management in the United Kingdom: A focus group study
Source: J Foot Ankle Res. 2024 Aug 21;17(3):e12047. doi: 10.1002/jfa2.12047 (PMC11339317; doi:10.1002/jfa2.12047)
Supplement: Supplementary file 1 — Supporting Information S1 [file JFA2-17-e12047-s001.docx]

**Supplementary files**

**Supplementary file 1: Focus Group Schedules**

*Patient Focus Group*

Before the meeting starts

When participants arrive at the meeting venue, they will be introduced to the research team. Refreshments will be available approximately 30 minutes before the arrival time if volunteers arrive early. Refreshments will be provided for approximately 15 minutes before the start of the meeting from the arrival time, or until all participants are ready to begin the meeting.

Participants and persons at the meeting

Lead researcher (Dr Emma Cowley)

Research assistant

5 patients selected based on the participant criteria in the ethics form

General Introduction

Greeting

Check the participants have read the PIS and signed consent.

Read out the ground rules for participation in the focus group.

Check all participants are okay to start the recording. Start the recording.

Emma Cowley to give overview of research and verbally check all of the participants still wish to participate. Remind the participants that this research is not conducted to assess clinical competence. There is no right or wrong answer today.

Inform the participants this is a semi-structured interview. Meaning that it is OK to branch out and talk around points raised from questions.

Remind the participants they can withdraw from the study as any time.

Protocol during the meeting

Comfort breaks may be taken as required.

Participants will be asked questions in turn, we will go round in circles until everyone has finished what they want to say, or until the conversation moves off topic, or until enough time has passed that the interviewer decides it is in the best interest of the data to move onto the next topic.

The meeting will take approximately 1 hr. The meeting will be ended if it extends to 1hr 30mins.

To limit risk of COVID-19 transmission, participants will be asked to wear a mask if they are able to. If they choose not to wear a mask they will still be allowed to participate.

Question guide

Questions may include but are not limited to:

1. What type of FOs did you have – custom, off the shelf, lab made?
2. What has been your experiences with your FOs – general thoughts?
3. Did your FO have any special features, for example, different stiffnesses?
4. Did you need to have any revisions?
5. Were you satisfied with time it took for your FOs to be made?
6. How did your podiatrist measure your feet for fit of the FOs?
7. What are your thoughts on that process?

Closing the interview

Check if there is anything else the participants wish to add

Check the participants are happy for the interview to end and the recording to be stopped

Thank the participants

Stop the recording

*Podiatrist Focus Group*

Before the meeting starts

When participants arrive at the meeting venue, they will be introduced to the research team. Refreshments will be available approximately 30 minutes before the arrival time if volunteers arrive early. Refreshments will be provided for approximately 15 minutes before the start of the meeting from the arrival time, or until all participants are ready to begin the meeting.

Participants and persons at the meeting

Lead researcher (Dr Emma Cowley)

Research assistant

5 podiatrists selected based on the participant criteria in the ethics form

General Introduction

Greeting

Check the participants have read the PIS and signed consent.

Read out the ground rules for participation in the focus group.

Check all participants are okay to start the recording. Start the recording.

Emma Cowley to give overview of research and verbally check all of the participants still wish to participate. Remind the participants that this research is not conducted to assess clinical competence. There is no right or wrong answer today.

Inform the participants this is a semi-structured interview. Meaning that it is OK to branch out and talk around points raised from questions.

Remind the participants they can withdraw from the study at any time.

Protocol during the meeting

Comfort breaks may be taken as required.

Participants will be asked questions in turn, we will go round in circles until everyone has finished what they want to say, or until the conversation moves off topic, or until enough time has passed that the interviewer decides it is in the best interest of the data to move onto the next topic.

The meeting will take approximately 1 hr. The meeting will be ended if it extends to 1hr 30mins.

To limit risk of COVID-19 transmission, participants will be asked to wear a mask if they are able to. If they choose not to wear a mask they will still be allowed to participate.

Question guide

Talking points include but are not limited to:

1. Acceptability of design plans of 3D printed FOs that we have tested

Design plans of 3D printed FOs will be shown to the group and participants will be asked for their comments.

1. Likelihood of developing a common dictionary of terms and the requirements for “old school” language”

The interviewer will briefly explain what we mean by a common dictionary of terms and ask about the likelihood of developing these.

Questions may include but are not limited to:

- How has the language changed since you qualified?
- What would be required to make this language common knowledge?

1. Software approaches to create a FO design with a digital prescription algorithm

Questions may include but are not limited to:

- What kinds of technology do you have access to, for example, an iPad/tablet for scanning feet?
- What are your thoughts on learning a new computer program or software if that was to be involved?

Closing the interview

Check if there is anything else the participants wish to add

Check the participants are happy for the interview to end and the recording to be stopped

Thank the participants

Stop the recording

**Supplementary file 2: Transcript from Patient Focus Group**

Focus group start time: 13:51

Focus group end time: 14:

Present: E.C. (Interviewer) E.L. (Field Notes) C.B. (Observer) P.W (P1) K.M. (P2) J.O. (P3)

Researcher introductions E.L., E.C. and C.B.

C.B. gives background to project

Participants introduce themselves P1, P2 and P3

P2: “I am riddled with arthritis, I have arthritis everywhere. I have done and do a lot of sport”

“I have had a couple of different experiences with orthotics, one good and one bad and that’s why I am here really”

“For me it started a long time ago, about 20 years ago”

P3: “Since I had those shoes I have always had foot problems … I blame those shoes. Since then I’ve always been mindful about my feet.”

E.C. discusses withdrawal of consent or redacting transcript

P2: “Ultimately, I want to scan the thing [foot] at home and have lots of pairs for walking, work, sport”

E.C. “We want to know your stories and your lived experiences with FOs.”

E.C. asks if everyone is happy to continue. “I’ve got a few questions which I will use as conversation starters. I’ve got a few questions put together to stimulate conversation, but it is semi structured.”

QUESTION 1: So, my first question to you all is tell me about your orthoses stories. How did you acquire them, what was the reason, a bit about why you’ve arrived at this particular junction?

P1: As I mentioned in XXX, I went to see several pods in Devon and they all have their different ways of doing things. Standing on something or with digital device lying on a couch but I couldn’t see how good that was if you’re not standing and putting any weight on your feet. XXX had me on my tummy and took pictures of my feet. But your feet surely change when you are stood on them?

E.C. What got you to the point of seeking help?

P1: Well I guess because of my back, previously I went down the stairs and hurt my back so I think that made my scoliosis worse so I noticed I was leaning to one side to the right and left foot not touching the ground. Because of my age I’ve lost flexibility in my feet and they’re a bit stiff so that didn’t help so I went to see a Podiatrist. I think really I should have seen physio to start with to get movement in the pelvis back.

E.C. Were your hopes with the orthoses particularly or more seeing a podiatrist in general helping you?

P1: Well, it was the podiatrist there but then they decided I was pronating when falling inward so lifted up my feet under the arch and I got forefoot equinus but I’ve looked it up and some believe in it, some don’t. I am aware especially with right foot that on the heel I can’t come off it so I have to have a heel underneath. Then you find the back of foot lifted up. I never wear very high heels but then you find it affects your toes as your toes are pushed up so that when I was having problem coming off front of my feet on toes more but didn’t really get pain in my feet.

E.C: Out of interest how did you come across pod as the answer to this problem?

P1: Well, it was several years ago and tv programme actually as showed gait analysis with sensors on limbs and everything and watching people walk. When go to podiatrist I found most had tiny little room with treadmill and told by 1 that you can’t see how anyone walks with the ground underneath you. You have to go over the ground to walk properly. So I’ve seen several podiatrists in XXX and then I came here and went into spire. Right foot problem because big toe wants to go lower than other toes. Its affected the rest of my body really. I’m going to osteopath because of my problems and I’m feeling more flexible which affects the way you walk as well because if I put my shoulder back and stand up straight I am higher. I can walk better with my pelvis.

E.C.: Do you want to go next P2?

P2: That was quite interesting. You try and fix the feet and then loads of other things stary to fall in place and you feel better. So having done a lot of sport for loads of years played squash at quite a high level for a lot of years my feet were getting more and more painful. In the interim I could get through it but afterwards when laying down have a rest get even more swollen and painful. So, whatever you do a little bit of spot, pain afterwards go with it but it came a singular moment went on to play a game but after 10 mins I thought I just can’t. The pain even with adrenaline I just can’t be dealing with it. It was just so painful at that point I had been to podiatry before as had a lot of nail problems as squash players don’t have very pretty nails as forces through feet because they come everywhere. A lot of arthritic changes with feet, especially with mine might because forces coming in unnatural way, turn feet in all directions so you crashing through, the momentum is going forward 2x2.5 body weight. If going forwards on court bang, the forces going in strange way, diagonally. Anyway, I foolishly finished that game but afterwards can’t play the next day. At that point need to do something more. Seen podiatry previously, had one removed nail grow back grow back, removed whole thing. Anyway, had a lot of experience with podiatry before but never thought about orthotics. Got one set, had some analysis done put my foot in the sand got measured up but weren’t very good at all. Arthritis got worse so I got another set which were much better. 2 different experiences, the 1^st^ set short, firm plastic and hard. Trauma from them reverberated through the body, I even got a retinal tear in year. Could just feel how hard they were and vibrated through the whole body. Now I have something that is softer full length and much more supportive and feels a lot more comfortable. So, 2 different experiences. hasn’t completely solved the problem but getting there.

E.C: P3 do you..

P3: I feel a bit left out from whole pod scene. I had hush puppies and I thought these were the bees knees anyway for about 5yrs after blue suede shoes I saw the GP and they said I had a heel spur and to take paracetamol and just get through it. I was making sure I was buying comfortable shoes. I used to wake up in the morning dreading putting my feet o the floor because I would have to start walking as then it was all good but as soon as I had to stop and rest then start again it was just awful. So I was working at Waitrose, drop boys of at school then opposite bus stop there was scholls shop and this one day I had 30 pounds and I knew I just needed something. My feet hurt. Went into the shop before work with my 30 pounds and said point me in direction of decent shoes. They said there was a lady at back of ship who looks at peoples’ feet so she might be able to help you. I said I’ll try anything yes please so my 30 pounds spent on a pair of insoles but same shoes but she advised these lovely leather ¾ length to put in shoes. Within 2 weeks my feet were sound it was like this was heaven for me the little lady in shop, came out with leather, was amazing. That was my very first encounter.

E.C Did you have others after that?

P3: They went strong for a very long time. I took them out of one shoe then put them in another and wore them for everything. That cleared up problem. Intermittently do have some foot issues but will buy gel pads and things as and when but those leather ones were the bees knees. I do miss them, and I do think about them now and again.

E.C. OK so I guess what I am interested in is the transition point for when you went to seek the help to when you thought orthoses would be the answer and how you came to know that. Was it the conversation/ preconception you were going to get orthoses and find them, when did the answer come to you?

P3: Just literally that little lady. I didn’t know she was a podiatrist she was just a kind lady. At that point with pain in my feet knowing I would have to do another shift just knowing I had to have something. A little angel.

E.C Serendipitous for you. How about you p1?

P1: Well I think I might have said when I realised I was going over to the right my feet not walking properly because not working properly toes curled up that when put in orthoses but toes decided didn’t like it very much.

E.C.: Did you find out during the conversation about orthoses or did you already-

P1: As I said I already said I saw a programme on television to see if I could find someone in Exeter. I didn’t realise my ankle. I needed something to support under my arch as I have high arches.

E.C: Did it feel like it aligned with you expectation?

P1: I was aware over several years it was changing my walking. The rest of body started to complain.

P2: I knew about the option for a while as when I saw podiatry previously they said you have a bunion here, but none of that was painful. I like to talk to practitioners. Non-stop questions. So when I got pain I said that and I might need some orthoses so I knew what I was getting myself into when I went to the first place for analysis and practitioner said you would benefit from some of these but like I said tried them hoping being optimistic but carried on but it weas bad experience. Then I went to have some injections across the joint but didn’t seem to make a difference. Consultant said come here as might get different options, that was better experience. Similar thing, get feet measured but test them, did get injured after using them but that was cliff edge, how much could I push the limits in my orthoses so you need to know sometimes where the boundary is.

E.C: Very Interesting point you just made. Expectation around orthotic therapy went in thinking might be part of solution but wasn’t from what I am gathering. P3 bit more comprehensive. So what happened, you had a good and bad experience, once decision made to have orthoses what processed different between the experiences?

P2: Process getting them about them about the same. Have a little look. I wanted to see the mechanics, see the skeleton foot I wanted to see the whole joint but I couldn’t really get it. So similar experience though here more technical and I quite like that. But after getting them trying them wearing them with 1st lot short, firm, high, slippy, they too high, instability, forces going all over feet, slide off of them, no give. My optimism kept me using them as you want them to work and that’s why I think sometimes you push it a bit more than should in overdoing it. Kept with it and got worse. You say part pf the solution for me it was part of the problem. It kind of exacerbated problem. 2^nd^ time much better same process to feet them but then trying it from very start felt more right, got chance to come back and adjust them if I wanted to. Bit high for squash shoes so when started playing squash again they offered to bevel them and lower. More fine tuning done with softer orthotics that one I had. I fact the 1^st^ ones did shave off but still didnt work. Similar process but as progressed trying them and getting back to exercise didn’t really..

E.C: Do you feel you had a role in process, a voice to help them understand what needed.

P2: Yes, I didn’t understood myself for a while the 1^st^ ones were.. I was already seeing consultant so he said see someone else so I voted with my feet as it were. Definitely, XXX said we can make them firmer, take a bit off bottom to make fit better. If tried them not quite right well adjust. That good its important to have that voice and give that feedback because things change all the time. You get your orthoses you do exercises it can cause impact elsewhere then what do you do you have to go back. See now I have plantar plate tear now my toes overlap but that’s not because of orthotics I’m just able to do more stuff. That might need extra taping tie to next one, thing evolve, if your active and pushing it a bit you need that feedback. Being able to print the thing and know I need a bit more in the arch.

E.C: P1 how about you. Can remember the process, feel heard in process

P1: Well, I kept on about being so stiff. Had a lot of massage on ankles and the feet but toes curl up so got annoyed with orthotic, lot for back and ankle but nothing for front. Made me annoyed. Partly why been to several pods but suppose toes flexible not much to do. Had pink tings used but skin sensitive didn’t like that.

E.C: That’s important as trying solutions get you a bit there but not quite then introducing new problem. Involved in feedback and what next step was did you feel you were part of? More done to you?

P1: Yes it was, I felt my feeling couldn’t get over to anyone

E.C.: What could have helped?

P1: Well I think I keep saying things. Right foot is toe dropped down kept saying need support on that side but couldn’t get across to anyone.

E.C.: Do you think that was in the moment or can you think of feedback could have helped.

P1: No I cant really.

E.C.: P3 do you mind if I ask you as leather out of packet. Only experience others since.

P3: Yes but only off the shelf like the school. Gel pads, self administered.

E.C.: As 1^st^ successful feel needed bespoke?

P3: I felt these were, I was in a lot of pain and I can remember she was kind and offered solution. I was quite trusting. I was young early 20s never heard of this stuff I was prepared to do anything to get through next shift.

E.C.: Sounds like quite short transaction here’s pain and solution.

P3: I worked through a lot of pain in those 5 yrs or so I had own business I had to be there. I couldn’t say I had foot pain so couldn’t go in. You do live with certain amount of stuff but that was unbearable.

E.C.: To go from that to no pain I 1 insoles.

P3: I think that’s why I remember it so vividly I had my leather things in such a shrine as it was just awful but within 2 weeks I was pain free

P1: Were you ever told what was cause of pain

P3: GP said heel spur. Chronic pain when put the feet to ground. Sharp needle going into heel, hideous horrible radiate into rest of foot. Standing feel need momentum to ease. When have to stop.

P1: Had any surgery?

P3: No nothing, worse period mid twenties so occasional issue aching put thing in there I have been very lucky when hearing you.

E.C.: You’ve sort of had a monotherapy where this therapy alone has helped.

P3: I was very lucky

E.C.: For you both you have tried things in combination. Do you feel foot orthoses at any point worked better with or after another therapy?

P1: I’ve had to bring my orthoses now for XXX to change up no I don’t know it is so much of it really

E.C.: I am curious long journey had injections

P1: I am lucky haven’t had a lot pf pain jut stiffness at my age and feel need to walk properly because of osteoporosis and keep everything working.

P2: I had injections separately, because first time didn’t get in 2^nd^ time more in but didn’t really help much so recovered from this so went for separate orthoses but currently changing running shoes as well as getting used to my foot orthoses and learning a lot buy feeling different running shoes make. My current thinking is something low to the ground not too high as foot orthoses not helping me. Lots and lots of cushioning heel I don’t think so. Not for me. I have wide feet, I need with in shoe for stability. I can’t come off and go over the ankle. That’s why 1^st^ not good. Going through shoe evolution. Other treatment ice, rest not really an option

E.C.: Transition from non-orthose wearing to now. Complex life, what was it like and how managed?

P2: For me difficulty is you only have 1 set so have to keep taking them out. Went for run on Tuesday and it rained and rained so they are so got so wet so you have to walk around in them either in wet shoes or don’t have them and feet get sore so what do you do? Good to have really efficient way to have more than 1 pair even if same pair printed but for me

P1: would be good to have 1 printed for every shoe

E.C.: and would you want same design for shoes as have different styles, height, different footwear for sport and work?

P1: As I said about forefoot equinus I need more heel.

E.C.: Ever had option for more pairs?

P2: yes, XXX said 2^nd^ get 30pound off

P1: Gave me a slimmer pair for nice shoes but I can’t have nice shoes as front of foot too wide. They don’t do smart shoes for wide feet. The whole foot isn’t wide its just the forefoot which is wide. My ankle is slim.

P3: Not tempted to get 2nd pair as didn’t have awful pain. Financially not afford but when great no pain even now I am mindful of shoes by. Very mindful as fear of going back to that, anytime I put a shoe on. I bought some flip flops and after 4 days it set off my Achilles issue and they go in cupboard hobbling trying different shoes, add an orthotic. I am very mindful. I don’t wear orthotics all the time just dip in dip out but left me mindful of feet and buy decent shoes.

E.C: Identity as people who wear orthoses do you see yourselves as that is your lot now or do you see as phase as get rid of or do you wat them for reassurance?

P3: For me I don’t wear fashion shoes I can’t bare, its dip in and out as and when so don’t need them everyday

P1: For me I am getting fed up I am wearing smart clothes but not smart shoes.

E.C.: A pair for dress shoes?

p1: I’ve even had a bespoke pair made during pandemic company in London came down to pattern of my feet weren’t very good.

E.C.: Stopping you wearing shoes you want. What would be required to get a good compromise?

P1: Bespoke shoes I have made for me from last of my feet but the top is too hard so as toes curl I can’t wear them I have to take them back, that frustrating. Big toe lower the others curl and spread.

P2: I have plantar plate tear so my toes are crossing over each other so if top is too firm then I need it to be soft.

P1: The arch has been lifted up on the right so I’ve just found out from chiropodist the nail is starting to ingrow so dig that out. You need someone to stretch toes out in right direction.

E.C.: To understand about the process hat would like to be added or taken away to lead to good outcome?

P2: I would have been good to have more time. You have your 15 or 30 min appt for fitting orthoses if fit ok your done. I would like to have had 5 mins on treadmill or test foot orthoses with sport specific movement to see if had a difference at the start because if I put squash shoes on and run lunatic might have discovered too high, too firm, too slippy for movement not straight forward and I’m going over my ankle. Whether that would have changed anything I don’t know because want to be optimistic hope will be a fix so go with it but I might have just been should have said too hard, slippy high. They might have said see how it goes but could have avoided issues but besides that process fine, wouldn’t take anything away. I think quite 1^st^ time put in sand and measured. XXX had an Ipad didn’t take long.

E.C.: Was it silicone sand that sucked the air out then goes hard?

P2: I think so, it makes am imprint one thing so he suggested a couple of things to do but coming here XXX had XXX who does feet strengthening stuff. I am starting to realise non-arthritic parts are weak, injured or also arthritic. Saw me couple weeks ago doing yoga and had to do a pose where you had to be on toes but found it very painful. That wasn’t the arthritis must have been something I hadn’t realised before. With Dave do things to strengthen feet and calves all around area that’s painful to reduce load or increase stability. From 1^st^ pod experience that wasn’t a thing. Rest for time, put orthoses in hope will be ok a bit too simple and limited.

E.C.: Have you had a conversation about mods over time or features or times you wear them?

P2: Not yet. Different pair for different shoe I went yeah squash shoe lower, running shoes firmer walking around. It could be a different pair for each shoe but definitely better to have more than one. I don’t think there is any going back feels much better with them. I can’t go barefoot makes a lot worse, suffer for days. You come to live with fact you need orthoses like a pair of glasses. One of those things don’t complain, it just is. Might be a better solution like contact lenses shoes printed with orthoses already in them. Maybe there is a better solution like contact lenses instead of glasses not an issue for me just something you have to do.

P1: You said about getting orthoses given to you they see you walking in them there and then. They said you away and say see how they go. That doesn’t seem right. My ankles quite flexible not strong but due to age.

E.C.: Maybe walk around in them?

P1: Yes

P3: I think you’ve made quite a good point with thing like your eyes. You test your eyes have the machine that takes pictures your feet are so important. If you have an orthotic and you think you need something. You think you need a dome thing or seething added or taken away quite easy with stick on stuff but if you’ve got something that’s bespoke and focussed on your particular foot you kind of feel should have time and space to say this is my orthotic it is giving me jip on this side is there something we can do to it there and then and have that time, as just an important part of aging and keeping fit and healthy isn’t it. I didn’t understand why it is quick put that in then off you go and come back if have a pain. It should be like going to physio or going to optician or dentist that kind of time and input.

P2: The whole thing especially with NHS you have dental check 6 and eyes retested I think even more important with feet as more important part of body but what you get in orthotic cam be changed overtime you change so even more so then your sight or teeth. If you decide take up new sport then -

E.C.: Part of health check?

P2: Yes then decide firmer, lower

P1: A lot of the people in my residential home are over 60 so sometimes they quite often fall over so I feel they all need orthoses

E.C.: Based on your experiences what would you like podiatrist to know about how it felt for you?

P3: I’m not sure, I don’t know if she was a pod but she was foot specialist. She was a gold star, right person at right time. It is having background knowledge to support someone like me and its that time and good communication being able to offer something I had been though about.

P1: I think several of them wear things in their shoes they have known about that.

P2: Like what?

E.C.: Like you’ve alluded to shared decision making. Interested to know how you felt about process, anything not vocalised at any time.

P2: I had MRIs, XRAYs so maybe if had time to go through that. Something look terrible but no pain so might not have been helpful but may have been helpful. I am quite vocal so we will be sat in there asking questions. I think to have more time to talk. It is quite focussed you’re in there, get measured, get stuff next time is it all ok yes then your done., its quite clinical and targeted. A little more stepping back, discussion like what’s your experience, what’s it like when walking, standing support might just draw out things that shapes what you get.

E.C.: Interested in contrast magical, mechanism and understand, and somewhere in-between, willing to try other things. As a podiatrist, how will we know what to drill down to if all of your expectations different?

P1: I remember XXX saying we all walk differently. Give you orthoses and you walk. they can’t say you’ve got to put that foot then as we are all different. We just walk as its comfortable for the rest of your body changes with way you walk, affects you, my neck is sore when walking. You see them as professional, but I feel like apologising when not working properly. You have to put up with a bit of discomfort and you think it is your fault.

E.C.: What’s making you feel guilty?

P1: Probably my personality

P3: The clinician knows all. At my age I see GP and feel alright but actually I know best for me should be vocal. I find it difficult to challenge someone who is higher.

E.C.: Inadequate collaboration. You have expertise I know what feels good and doesn’t but something missing in middle feel not quite there and can’t vocalise. What missing?

P3: It is tough one. You are going to someone who specialises in something they can help you with. I am coming and paying for someone to help so you are expecting knowledge. But it is tough as in one respect don’t want the to be best friend must want their expertise to give you answers or support

P2: I want to know why. They are the expert, you come, they give you something and say on you go but you might get chance to come back. I want to know why it is going to help 1^st^ time it is going to make your feet higher so you won’t be bending so much as bending causing problem. I would have gone with that bit actually I don’t think that’s the issue. Now I know a bit more about it, I know the why and time to discuss and we both confident got right solution. When things feel like going wrong, I can say is it because I’m now in high shoes less stable so is it instability the problem or is it I’ve got carbon plates in my running shoes. Just to try understand what this is going to do. At clinical level got pain solve it, the mechanism by which it does that, thinking of doing another sport I won’t because I know the why, just going to the stop it doesn’t do it for me I want to be more involved in it.

EC asks anything additional needs to be added. Thanks to participants.

P2: Be interesting to see what you do with it and how it goes.

E.C.: I do have 1 question about expectations/, mindful might come back. Do you think orthoses counter that vulnerability?

P3: yes Probably, for me, if something happening, fairly confident as had good experience it would help.

E.C.: high faith

P3: haven’t had bespoke, no foot issues that I did would go bespoke.

P1: I think they have helped more secure with balance.

P2: I’m fearful that foot issues will be limited factor still being painful is my worry. Foot orthoses are part of broader solution with shoes and exercise and strengthening. Coming here is holistic approach. Talks a lot about shoes rockers, plates. Part of broad set of solutions not going to go away the arthritic change not solved but not be a limiting thing that’s great. No magic/. Not too be the thing that stops you.

E.C.: Thanks to participants, form given

**Supplementary file 3: Transcript from Podiatrist Focus Group**

E.C.: I'll just reiterate a little bit of what we've just said. Is it recording yet? Ohh. There we are. Lovely. That's super. So we just, just for the sake of the recording, we're just doing introductions and we're expecting XXX to join us in a little while. And as part of this evening, we're just talking through the introductions of the people in the room and then we'll talk a little bit about the work package as well that we're doing after that.

J.B: Yeah. So yeah, my name is XXX, I'm MSK. In sports podiatrists working for XXX NHS Trust, the NHS. And then I also work in private practice as well.

E.C.: Brilliant, thank you. And just as a quick one, when did you graduate? How many years experience have you got?

J.B.: So I graduated in 2016 from XXX and then I've just finished off my Masters in Sports Exercise medicine, paediatric sports medicine at XXX. Thank you.

E.C.: Alright, that's brilliant. Well done uh. I'll go anticlockwise around my screen and XXX, your next please.

C.D.: OK hi, I'm XXX. I graduated from XXX in 2003 and, I've had a private practice and run an orthotics laboratory for 19 years and now working for Solent NHS Trust. And I've also started a private practice and orthotics laboratory here too.

E.C: Wow. So you've got lots of experience, industry experience as well as clinical. So I'm really hoping we'll get some good insight from you. Thank you. XXX, you're next, please.

J.W.: Um, I'm XXX. I'm working in XXX. I'm currently working with the students and apprentices, but if I had a lottery win, this is what I would do.

E.C: This is what you would do. What come to focus group?

J.W.: Um, yeah and um biomechanics and 3D printing and. OK.

E.C.: So you've got really keen interest in this.

J.W.: Yeah.

E.C: But, but I'm guessing given the NHS in terms of its provision and opportunity and orthoses, you may have been forced down a particular track and may not have some of the experience, for example, in some of the areas of orthoses that you might have had in private. Do you think that's true?

J.W.: Ohh, absolutely. But my background before this was sports massage therapy and rehabilitation of injuries. OK. So from the practical aspect of it, I've got a lot of experience. Yeah, it's, you know, brilliant.

E.C: Thank you. That's, that's fantastic. Can't wait to hear from you as well. And last but not least, we've got XXX, please.

N.G.: Hi, I'm XXX. I qualified back in the Dark Ages in 1990. Been working in biomechanics since 1999 in the MSK Primary care service in XXX when it started in 2003 and have been the by mechanic service manager since 2007, something like that experience. That's great donkeys’ years. And with the 3D printing, when this was first mooted many years ago, I went up to XXX to Peacocks Medical Group when they had their 3D printer and the project work that they did with that and they had this specialist come over from BMW because that's where they learned to do the 3D printing from, right? That's great. So.

E.C.: It's so lovely to have such invested people. Uh, XXX is bang on time for just the last introduction as well. Evening, XXX. Evening. How you doing? Just been around the room doing intros and I'm afraid it's your turn straight away. So can you give us a quick intro to who you are and what your experience is with orthoses?

N.K.: Yeah, my name's XXX. I run a little clinic outside of XXX called XXX. I would say 90% of our caseload is MSK. Based use orthoses on a regular basis, most probably about 30% of our caseload and not the foot authorities. Anything from form toxics to 3D printed or carbon linings.

E.C.: Wow. So a really fantastic diverse experience there. And I think amongst us, we'll cover some good ground this evening. So I'm not gonna be shy. I think we should get stuck in, actually. And so the background like I said, of this project is about understanding what podiatrists do, what they what their frustrations are in the process of prescribing for authorities. We're starting, XXX, just you were you weren't in the room. We're going to start from the point where we've decided that someone needs foot orthoses. Like the process that happens after that moment, so not the lead up to when someone needs them. Although unless it's relevant to the process we can dip into, that's just to focus the conversation a little bit. So I'm interested to know now I know a little bit about you, what you feel about prescribing foot foot orthoses in terms of confidence in yourself, confidence in the process, perhaps the process you were taught or the process you've learned since. How do you feel about the whole thing of prescribing for those sees? Um, so like kick off then go for it.

P1: So I obviously work in two very different areas. So for the NHS I have to write up, I take casts or foam boxes of people's feet and I have to write out a prescription form and then I get the device back. Currently it takes about 8 weeks. I have absolutely no faith in this process in that the what I prescribe is not coming back and it doesn't it really many of the orthotics. And they come back, have to be redone or refitted. I think there's a real communication breakdown. On the other hand, I have a private practice where I have my own orthotics laboratory which I have 3D machines and there I have complete confidence that I know the person is going to get what I meant, what they need. So it's two very different things.

E.C.: OK, so your primary concern when you're forced to use an external lab is the disconnecting. In communication and interpretation of what's going on at each end, is that fair to say?

P1.: Yeah, I think that's what I've found anyway is that pretty much. Almost any prescription form is that you're essentially giving away your intellectual property to some person on the other end who you lose that connection. You know they always say biomechanics is a mixture of science and art. And I think that you lose an awful lot of the art by letting someone else onto your turf. And certainly when I'm, when I'm making an orthotic, I kind of just somehow there's a little bit of extra that I just know that person needs that I can never communicate. To a lab, no matter how hard they try.

E.C: That's fascinating. So language itself is a problem. It's the words that we have available to

use.

P1: They're just the the vocabulary is insufficient. But yeah, yes, I think, I think the forms are, you know, the prescription. The problem is that despite the loads and loads of orthotics labs and lots of work that's been done in trying to design the perfect prescription form, I don't think that exists. And uh, either they're too complicated. It's almost like they want to take the job away. You know, you see prescription forms that will say where's the pain, what's the condition, what's this? It's almost like they want to make the orthotic the way they want to make it and then you get the one we have at XXX NHS Trust with with the our orthotic company is that it's like orthotic material posting cover done. And so it's incredibly primitive. It's complete potluck as to what you get back.

E.C: Thank you so much. That's a great start. XXX, you were going to come in just a few seconds ago. Did you want to add to that?

P2: Yeah, I I agree with P1 is that the the issue is, is. The big issue current I feel is if you look if you've got every manufacturer’s labs form. All the language is different and. Also, if you ask if you wanted a 5 degree medial hill post and you got every lab to make you one, everyone's 5 degrees is slightly different. So actually I remember when I was working one lab, he's like what's the point to me writing a number he says I'm just going to write small medium and large and it's not ended up doing and it is like there is definitely that blend you know, it's blend between art and and science and the it's that communication. So This is why I'm I only work with set number of labs and I don't care another lab would come back and sell me a product for half the price. I won't move because that lab knows how I communicate. And even then, sometimes I don't even use their lab zone form. I write it in my own language and they know what I mean. Which is OK for me, but that's not scalable at all for a profession because only I've got my brain. No one else has access.

E.C: So the confidence there is in for both you and P1. I'm getting and I'll come to the rest of the room in just a sec as well.

P1: Um, that you have more confidence in yourself than the lab you're using. And it's imperative that you build up trust in the lab so that you have confidence or at least more confidence in them. It's something like what you would do yourself, which works wonderfully until that particular technician that normally does your work decides to retire or years. So, you know, obviously none of us get it right all the time. But I think in working in any field, you're gonna have to have a certain amount of confidence and obviously sometimes misplaced. But at least I want to know that I'm going to get what I've ordered.

E.C.: OK. Thank you.

P1: I think to add to what XXX said there earlier, I mean one of the things we use is a PMP. And I mean, I think the the current form we use says it says the PMP and they there's no way you're going to put it. And if you for us biomechanical podiatrists you know that. Something like a PMP is an incredibly subtle and powerful tool that can be make a massive difference or just be a painful lump under your foot. Exactly. And it's critical that the placement of the shape that the the thickness, these things are. Or everything that make or break absolutely the Marmite.

E.C.: Right. I'm going to come to the other side of my screen now. So you two, you've, you've had your opening session, which is wonderful. I want to hear from the other three just quickly and then we'll bring it all back together. So, XXX, you were nodding vociferously, vigorously during that. Do you want to come in at this stage?

P3: Yeah. I mean, we've been down here in XXX, we've gone through three different labs in the past five or six years and we used to have our own lab which got closed down. I won't go into that because I didn't agree with it. We've changed labs say three times and that has been out of our control because the way XXX Healthcare works, we are working with some of the orthotics departments as well. And so the contract was put out to cover both aspects of it. And so contracts were changed for nefarious things like they wouldn't deliver at a certain time to a certain place. Forget that the quality of the orthotics was actually not too bad. I mean I agree with the others that it is. A lot is down to the lab interpretation. But once the lab gets to know your language you do get much more consistency of devices, but you you get variations amongst clinicians and and what I ask for and what my colleague asked for although we think we're asking for the same thing, if we use slightly different terminology you'll get different things back. So there's a lot of factors you know that affect the orthotics and how we get them when they get them and say we've had three changes. Of supplier in the last however many years. Um, yeah and it's not been great.

E.C: No. Is it not that great for you do you do you still quite confident in yourself that you know what you want it's just unfortunately the other end is changing is that is that knocking your confidence too or is it just frustrating you?

P3: I think it it's frustrating me and it's making it very difficult for the less experienced podiatrists to know how to have confidence in their own prescribing because they're getting things back and when you compare because obviously when they when we first started changing we would check the prescription against what we thought you know what we were physically getting to make sure the material is white and it looked about right because obviously we hadn't seen the individual patients. And to a certain extent, they were being made to the prescription with no thought about how it might even fit in a shoe. And some of them were coming back like canal barges, you know, ridiculously big. And and that was a huge, huge problem because most of them were going back to be remade. Yeah but that then simplification as well absolutely not. Not just for us but for the manufacturer but say that would knock the confidence of some of the more recent yeah recently qualified podiatrist because she doesn't get their insulin isn't it. So yeah, precisely the patient as well.

E.C.: So yeah, it's there's it's multifactorial really it is that's what we're gonna uncover tonight. I hope it's this is about the conversation is and can I just come to either XXX or XXX just seeing as you haven't had your say just yet. So uh the things that you like and the things that maybe you know your confidence in the process and in yourselves.

P4: Yeah, I mean, I would echo what's been said already in the sense that I think communication with the lab is paramount. Um, I think I've used a variety of different labs. And I think that it does take a little adjustment period to to, yeah, get used to what their prescriptions like saying like the, say, the terminology, which is different between different manufacturers. And I find that it normally takes, yeah, that adjustment. And then I usually say you get to a point where you do feel a little bit more comfortable and you feel like you have a better idea of when you, you know, send off that device, what's going to come back. And you get fewer surprises with that. Um, I think that something that I've learned over time with for orthoses and again and I would be interested to hear everyone's perspective on this as well. But I don't think there is a perfect prescription for any individual. I think there's many ways of trying to do the same sort of sort of thing really. I see, you know in terms of mechanical aspects at least that they are load modifiers and you're looking to offload certain tissue and then you're going to most likely, yeah well, you will increase low through other tissues. And so I think there's many of different ways of. Doing that and like I said I think that you know and as you know and I'm sure we'll come on to soon. We're given the topic that we're talking about you know with 3D printed devices and with lots of other new devices that are coming out there. Again lots of different ways in terms of modifying shell density in different areas and you know the cast dressing and all sorts of different things that you can do your clothes. So you know you could come up with two completely different designs of the devices but ultimately you know they get the same outcome and I think obviously with certain people the therapeutic window is quite narrow and others actually it's. broad. And so you can get an easy win in some cases whereas others like say you really you know got those fine margins of what you need to the device to do. So that would be my, my thoughts on it and like I say I think that yeah my my confidence grows with a particular manufacturer as you've worked with them for a little bit longer like we've touched on already as well that I think that you know if the the staff who work there change or the management changes or they make cost savings or they the way they do it they're not. Also I found with certain labs as well that they're really good with. And shell materials and then others they're not so great. So they're calm fibre device might be really good but then their EVA might not be so good for all you know vice versa. And so I think the understanding that with those labs then I can manipulate my prescriptions accordingly to hopefully get what I think to be right. But then again the other sort of nuance probably where the art comes into to what we do is that we don't know for sure exactly how patients are going to respond to devices. And even after we've had got loads and loads of experience and we've done lots and lots of devices, still sometimes a patient comes in and. Was a lot better than you might have thought. They might do a lot worse. And I think that's because again you know, it's, it's a, it's a complex area. The way the central nervous system responds to it, people, psychosomatic response, all these other facts as well as just the, you know, biomechanical facets,

E.C.: I think you've got to be taken into consideration as well. Yeah, thank you. What a great start from you. Thank you, XXX and XXX, have you got any little pearls for us?

P5: No, I mean I work in a different part of XXX to Nikki and we don't have access to the same things. So basically because um. If I can't make it out of poron. It doesn't happen. It yeah it it gets referred on basically. So I would, I would do everything that I could to kind of make it out of power on and test it. We have the facility to then do like a cardboard template and send it off to. A lab, but in all honesty if if I couldn't do it out of poron then I would refer it on because what I was getting from the lab just. It just wasn't what I wanted. So um. Just basically do most of it, or did most of it myself and we started to get some more robust shells in towards the end. Uh, which could pad out a bit better. But yeah, it's mainly proof of concept out of poor on it worked or it didn't. Right.

E.C. That's really helpful. Thank you. So just taking us into the next question then I'm going to just be force you to tell me what you like about the process because we've expressed a lot of frustrations and I think I've heard some of what you like when you get a good relationship, it works quite well. But without that relationship, it's, you know, potluck almost. What else do you like about the process? Uh, where do you feel that it works for you?

P5: OK. Can I just so, so from somebody from the back of beyond? The forms are quite good because they do try and make you think and they kind of give you a tick list to go. Have I missed something? For somebody that doesn't do it every day. Yeah, obviously you guys do it every day.

E.C.: That's why I think you’re your opinion is really valuable, Jenny, because I need to see everybody across the spectrum as well. So I'm, I'm listening definitely. Is there anything else that you find particularly helpful? Is that?

P5: Oh, no, I'm done. You're done.

E.C: OK. Go for it, Chris.

P1: Yes. So I think that what I'd like to just to kind of tell you with the way I do it, it the way I like to do it compared to the way I'm forced to within the NHS. So when I got involved in CAD CAM technology but eight years ago, So what I got was the whole process, the machine, the software. I designed the orthotic myself. You know, it doesn't go to the lab. It's and it on CAD to design an orthotic is you can make that thing perfect, well, perfect. What do you consider to be perfect, which might not be perfect, but you can design that thing. If you're going to put the Marmite on your orthotic, you can shape it, you can cut out, you can look, you look at it and go. Now that's what I want in the position I want for that. Thing. And so when I'm happy with the design, I press play and the machine makes it for me. So essentially and also kind of wearing my hat as running an orthotic lab, when you're making for someone else, the moment you have, they provide you with the ISO file, the digital file. There's no argument about whose fault it was if it didn't work because they designed it. And for me, I'm I my way of making orthotics is that I will design it Millet in the machines I have, and the patient gets it four days later. And it is. I can't imagine doing it any other way, quite honestly. I mean, it's tricky in the beginning, you know, you're going to make a lot of mistakes designing it, but. Once you've designed a few, it's it really is. There is. It is unbelievably simple, and I cannot imagine filling out a form privately ever again. Quite honestly, yeah, yeah. Everybody else. Everyone else got things that they like about the process, but it works when it works.

P3: When it works, it works well. I mean, we we're fortunate, I suppose most of the time we get our devices back within four weeks. Which is comparable to when we used to have our own lab. I mean, we had a CAD CAM machine. Don't think about. It's about 15 years ago and it is great being able to work on the machine and and design it you know as XXX does because what you see is what you get and having our own lab made life so much easier because if if things didn't work you could get the alterations done quickly. With outside labs it's much harder but when everything when all the Ducks line up is perfect.

E.C.: How about you, XXX? What do you like?

P4: I mean, I think that for me, so again, I think someone else touched on this before as well. Obviously there's lots of different ways of of taking an impression of the foot, whether that be, you know, with phone impressions or with plaster of Paris or with 3D scanning. And I do a mixture of those at the moment. I'm trying to shift everything over to doing it digitally. So the moment I do just in private practice the 3D scans, but bring it into the NHS as well. And I find that that does adjust my prescriptions. Is slightly with that and but I enjoy the process regardless of which way of around it is. But I find that um yeah then they give you a little bit more of a challenge because you know usually when you do the impression box it's either you know full weight bearing or something weight bearing to a certain extent even if you're pushing it down with your hand. Whereas yeah you can do it completely non weight bearing with a plaster of Paris or 3D scanner you can manipulate the foot as well, slightly as well. And so I think that then I have to make an adjustment with my prescription as a result of that depending on what I'm trying to achieve. There and again you know with varied success and again it's a learning process with each one of those and I probably initially you did a lot more work with foam impression boxes. So I got customed to using that whereas now I say I'm trying to transition away from that just because it gets a quicker turn around more environmentally friendly and also you getting a very accurate impression of the foot as well. So yeah, so we're trying to move towards that. I actually enjoy that part of it and obviously you know I like you know a big part of our job is enjoying it when hopefully when things go well and when someone comes back and they really, really happy with them and you know. You're, you're, you you've achieved their goals. And so yeah I I I enjoy the process of it and you know I, you know sort of nerdy about when it comes back and how I like all the other pieces that come on to it and everything else like that. And so yeah no I do really enjoy making devices. But I would be lying if I was saying that that you know that was that smooth and that great every time. And so you know, it's still always there those challenges there and I think I get most frustrated. Yeah something does come back that isn't what quite what I had imagined or that you know the patient has responded differently to how I'd imagine and again that was. That's got a learning point for me and it might be that they, you know, they, I don't know, do it like the activity they do is a little bit different to what I've worked with before and I'm just having to learn about that or you know, whatever it might be. But no, I generally enjoy the most of the process and then yeah, like I say, when it obviously when it goes well, it's really, really great.

E.C.: Listening to you all, and you know, I'm just remembering the heart sync moments over the years when you pull it out of the packet and you're like, what is that? Yeah, that's not what I asked for. And you just know there's a whole lot of drama. You got to have a difficult conversation with the patient. You got to send it off. What exactly? If you ask for it, will they change it? Is it even going to be the thing you asked for it? It's just a heart sync moment, isn't it? But anyway, I'm coming back to you because I'm. I don't want to talk too much. Nick, what do you like about the process?

P2: Um, it it. It's all joking and saying I like it when it works because for us I would say the amount of modifications we have to do are very minimal because I think it goes back to building that relationships with. With the labs. So yes, I would love to design my own mate, the modelling clinic and issues. We've looked at it, but just from time and economics, it doesn't quite stack up. Um. Yes, yes, but I like that concept. But at the moment I like that relationship that you've spent time building because I have had arguments where I have phoned owners of labs up and said look, this is a pile of XXX you just sent me. Get your ideas up and I want this sorted. Now type thing because it's just and yeah it's it's that, it's the process of that. The digital scanning again I've been, I've been doing it for six years now using the structure sense scan again that makes my life a lot easier. Patients like that experience as well because they think it's new technology when you tell them actually it's not that new and it just adds a bit more theater to that patient experience and then they come back and they see their foot and they're Oh my God, that's my foot. And then they get the device back and. It it it all becomes that nice journey for the patient with it all then hopefully goes the plan. Don't get me wrong, I remember it. In my NHS days. A patient coming for review and said, oh, the authorities are wonderful. They had the left one in the right shoe upside down, yeah, upside down. And they're like, these are wonderful. And I'm like, well, where do I go from here? Nice, yeah.

E.C.: XXX, you said you liked the forms. Is there anything else that you wanted to add before I move on?

P5: Um, only that I mean, I went probably about four or five years ago now. I went up to it wasn't, it wasn't orthotics lab that we dealt with. I just happened to be at the XXX conference and I got talking to an orthotist and she said, do you want to come and see our factory? So I went,

E.C.: yeah, OK, brilliant.

P3: Um. And I don't know if it's standard practice, but in that factory they would take the foam box, they'd scan in the impression, and then they would flatten out all the lumps and bumps. And I'm like, but what? What is the point? And they're like, no. If you want the lumps and bumps, you have to do a cast. OK. And to me that just seemed like a. Massive missed opportunity.

E.C.: Sorry, what did you say, XXX?

P3: There's different ways of modelling the CAD CAM, if it's if it's the one I'm thinking of. That you went to, it's possibly their standard practice is to smooth the impressions. Yeah, regardless of what you asked for on the prescription. But you don't have to do that. The CAD Cam will let you do lots of different manipulations with. It's great fun to play with it.

P5: Yeah, but that might explain some of the results that I'd had in the past. So this is this is bringing me forward again then in the conversation you honestly .

E.C.: We could spend hours talking about this we you'll have to just put up with me when I move this on because I want to break the mold a little bit and think about you know we've thought about our frustrations. We've thought about what we like and what we don't like. XXX you've even gone so far as like making up the whole process and setting up your lab so you're in full control of the situation in at least one of your roles. So that brings me to what would you change? About the process, that is sort of the standard industry process at the moment. Um, if you didn't have your own lab, but I guess drawing on your on your experience of setting that up would be absolutely valuable for this conversation as well. So what would you change?

P1: Would introduce um CAD CAM design at university level. And if you work with a lab, they must give you the software so that you you can take. I believe with authorities that there is no right. There's only two things in orthotic must do. It must be comfortable and it must work. There's a lot of argument about this material, that material, this technique, that technique, I don't really care. It must be comfortable and it does work, you know, that's the bottom line. Now if with a lab, whatever technique, if they say to you, OK, cool, you want to have your orthotics made by us, we're giving you the software. And there's companies that I have worked with before where in XXX where they will give you the hardware and the software and you commit to sending them a minimum amount every month. They train you on how to use the software. So you design it and when you send it and you e-mail the file to them and they mill it. So it's your orthotic. And I think to myself, I haven't really seen this yet. It surprises me. I'm not seeing a lot of podiatrists that are sitting down doing the CAD. And I, if I could change something, I would say teach it in university. And when you go with when you start working with a lab, they need to give you the software so that you retain your intellectual property because we are giving it as podiatrists, we're giving it away, we've got a technician. You don't even know what training they have. They just don't know who it is. And I think that taking we need to take back control,

E.C.: That's what that does happen in the Australian schools. So I did a visit in in the summer and I asked them about this this process and they do exactly as you say. I'm guessing it uh, there's the XXX school. Am I right in thinking it's not open anymore?

P1.: No it's well it's it might as well be open but maybe it might as well be closed. I think they still struggling on but in XXX there's pretty much one company that's taken over the whole thing and they make. Well, there's only like 120 podiatrists in the whole country and I think they move for almost every one of those podiatrists and essentially they you do what they call a million contract. So essentially they give you the software for free and all you have to do and in some cases the hardware for free. All you have to provide them is a minimum amount of orthoses from their company. And I think it's really few, I think it's 5 pairs a month and they'll give you the software and the training and actually to be we're now very deep into the process of bringing that to XXX NHS where we're going to be using that system which is and we're going to be training all of our band fives that are interested in in MSK to do care themselves, which is to me very exciting.

E.C.: Actually agree? Um, how about uh, XXX and XXX? And what would you change?

P2: I'd I would echo what P1 said really is, is that? It's the grassroot education. It's education at the undergraduate. Level and there's also the process of how we design. I still, I do believe that we should be the ones designing. We're the one who's got the foot, the patient in front of us, we're the ones know exactly what we want. And I think we al agree the biggest issue we have is to breakdown the communication. And that's just because we're trying to write down on a bit of paper this 3D living bit of tissue and say I want you now to do this for this person that you're never going to meet. You're never gonna speak to you. You're never gonna know how they are, how they react to things or anything. And I want you to design this. For me and this sort of like you would never. You would never work in any other industry like that and I think we need and with technology you look at the likes of what's the Dutch is it volex care I think and they mill for a lot of the big German companies like Bauerfeind and you can buy software from them where you've got to have no coding experience. You literally do like a drag and drop and you say I want this and it will show you OK if you do this you're orthosis is now going to look like this so and then that leads into the next thing is. The universal language of what does a PMP pad look like? What does a first met cut out look like? So actually, if you ask of something. You get a standardized item back.

E.C.: P4 anything to

E.C..: Ohh, sorry P5 do you mind if I just go to P4 quickly and I'll talk to you in a sec.

P3: Yeah. So I I think that I think what we said already about having CAD CAM designer at university would be really valuable. I think that would give people a much greater sort of depth of understanding of how these devices are made and again you know how the device might return and what they might be thinking about with their prescriptions at that level. And then I think that then a guy totally agree as well I think. Only a consistent language. Across the board would be really, really useful. And I think in an ideal world, I mean I think this will probably will become viable at some point. I mean hopefully not in the too distant future where you take the 3D scans there and then in the clinic you then have your own 3D printer and it just prints out there for you. And then I don't know how long it would take, but then you could just have them all done there like say all in-house. You're making it exactly how you want it. You can you know, do the CAD Cam adjustments or yourself and that's it. And like you say, you're hopefully, you know, mitigating. Some of the risk in your, uh, controlling some of the variables like I say, of going through the communication through other people and all those other things. And you've got a lot more sort of control over that. And I think that would be an ideal situation. And like I say and I don't think that necessarily is too far into the future, but I think that would give the best patient outcome as well in terms of consistency of device, quality of device and and then also there's return being so much more so quick. Yeah. Yeah.

E.C.: Thank you, P5.

P5: I might be way off base on this one. But just some systems do not send the 3D image that you've scanned in to, for example XXX or XXX, because I thought these companies did that and they then used that to help build the insole so they could mash the two together.

E.C.: Do you mean the image of the orthosis? No the image of the foot?

P5: The image of the foot, that's right, yes. Yeah, they do. So, the scanned file, um, goes. So, if they have with a paper prescription normally, yeah. So, if they have the two can they not and I thought that was the process that they could, they could put the image of the foot with the orthotic. In which case there shouldn't be too much. Disparity.

E.C.: So if I can and so if P4 or P2 could

P1: correct me if I'm wrong here, but from what I understand from the process here is that you send them a, a version of the foot. It can be a foam box, it can be a digital scan, it can be a force plate measurement, it can be a plaster cast. When this thing arrives at the lab, they scan it into their system. They're either they don't have to scan it because it is already a scan or there's a phone box, they scan the foam box if it's. Plastic cast. They scan the plastic cast. They then looking at the prescription sheet design on CAD, the computer aided design, they then design the orthotic. They then interpret what you've written on the prescription form coupled with the foot impression that they have and they make the authority correct and what, how, how are they getting it so wrong? The opinion and I refer interpretation, I think, I think that's where it is. And I think, yeah, again, you know, the way that I would make a device must be different.

P4: P2 did over to P1 to you journeys and P3, you know we all have slightly different preferences and again because this is not our, you know, we haven't got. The science and the data just, you know, just to analyze everything and throw it all together and say this is gonna be the perfect device. There's, there's, there's, there's a human aspect to it. And I think it's just counting on our experience, understanding the principles of understanding of that individual from the time that we do have with them. There's so many different things that come into that. And like I said, I think my understanding is that there is a sort of like say a therapeutic window and it's just depending on that individual, you know, that could be really, really like say easy win or it could be really, really tricky and some people you might never win. And I think yeah I think that is sometimes you have to just put your hands up and say you know that's that's just and that might be related to their expectations and their beliefs and you know all sorts of different things. So I again you know it's not just the mathematical equation either. So I think that and how do you put on a prescription form saying that this person you know. They've got hyperalgesia or they got they got central sensitization or something like that. You know we don't write that on their normally, but that is still something that's very much relevant to you know our prescription. And I think also some part of the other issue is sometimes I think the people sending in for 4th this is don't know what they actually want. Yeah I was gonna say this is the issue isn't it?

P3: Yeah. The other thing as well is that a lot of these labs were set up with orthotists in mind, not podiatrists. And so we are sending, you know, although we do very similar things, we are sending things with our language. And the labs are used to working with the Orthotists language, which you can say the same thing, but it means two different things. Depending on the authentication gap, yeah, because they're looking at you. If they've had years of experience of working with orthotists, they are tuned into to what their interpretation of that particular terminology is, which is different to ours. So I think if you know if we could change one thing that if we're going to continue with external labs, if we could have these labs run by podiatrists for podiatrists because then we would all speak pretty much the same language with. Little variances you know cause all the schools train in slightly different ways, but I think that would be probably one of the one of the biggest benefits that we could have and again as the others have said, training the students in scanning and 3D stuff.

P1: To be fair to orthotics laboratories, to just on that side of the equation, I did an internship with a lab years ago, someone called XXX. Well, you spent person and I then also ran an orthotics lab of my own in XXX. And I have to say some of the casts that arrived, they don't even look like they've been taken from a foot. That's right. They, you know, people would take a course and it's still with they're putting it in an envelope and send it. And he's like, how on Earth am I supposed to. And he would essentially take that cast and kind of try to imagine what it was and then look at and try to design like read the podiatrist. Mine to try and design it so and they don't know who's sending it in or what. So there is a real I think in this within the industry I've always been very jealous of opticians. They get it you know they it's so easy the computer tells them the the prescription, the machine makes it and it comes back three days later and generally they work and we are dealing with it. It's a much harder and at the same time you get someone who cheerfully pay 300 and. £5400 for a pair of glasses that cost them £20 to make and yet a payable closes. You tell them it's going to cost them 5, four, 500 rands. They they blank out on you. So you know, orthotics are always tricky to make. They're always expensive, they're always time consuming, and in 20 years I've I feel that I kind of found something which is it's once you've paid for the machinery, which is a it's an outlay, but the they're inexpensive. To make to Muller people sonics for me, I mean I can make them. And in in XXX we were offering a next day service regularly. And and then accuracy. If you get it wrong, it's your fault. You know it's not someone else. And that's what I use now and I can't imagine doing anything else. But that gives you a reflective. It actually commands you to be reflective because you're forced, you know, to be accountable for your decisions aren't you? Well if you if you design it and it and if the machine molds it, you look at it and go oh, did I really tell the machine to do that or tell to do that. And I mean if you're designing. And you're sitting with a machine next to you and you design something and you Millet and you go oh that's what it looks like and then that's how you get better and but it's a tricky business you know it's not easy and I think that orthotics companies are trying to actually push podiatrists out of the process. If you look at these new fits orthotics this is a company they used to be re scan and that that machine is writing your prescription for you. We've got podiatrists that are that are presenting those off. And I'm going what about if the patient, you know, this thing does? There's no subtlety here. There's no, it's really just a does it have electoral wedge or a forefoot wedge? There there's no subtleness that goes into a really good orthotic for a complicated foot pathology. They want to take us out of the process.

E.C.: Yes. Yes. And so you're speaking so much to my sense of vulnerability here, XXX, because I'm sure we've all been there. We've all felt that moment where, you know, you are a little bit tempted, truth be known, to tick the box. Sometimes, you know, go and you decide. At least in my earlier career, I was. And then I got to be a complete control freak and wanted to know exactly what was going on. And this is when my research journey and my experience increased. And I ended up because I was in private. Practice. This wasn't when I was in the NHS and NHS you worked with XXX and his labs a lot, but in private practice when it was just me, I started beta testing. So I started molding up um from, you know, low profile foot orthoses using poor runners XXX does. Or anything else, any sort of padding EVA that I had a, you know, a grinder in the kitchen, I would you know you know, grim old days grinding stuff off and building up the orthosis to the profile and the attributes that I wanted in it. So I knew exactly where that Dome. I knew exactly how much that arch height was. What I didn't and could never know was the, you know, the newtons of force I was applying because no one ever knows that. But I could get a feedback from the patient is that too much? Is it was like lens one, lens two in the in the in the optician sort of model. And I'm just thinking as you're talking in terms of we're breaking the mold a little bit here in conversation, it doesn't matter. It's a safe space. Um, I'm tempted to say in, in terms of building confidence in the junior podiatrists and, you know, helping them to get to know what works and what doesn't. Is there any merit in building a sort of a beta device and scanning the device because you know it fits in the shoe, you know it works on the patient, you know roughly how hard it is. What we do is we scan the foot and like you say XXX, that gives the decision making to a certain extent to the lab, but if we scan the device that we made in 3D. We've got a 3D device. The patients walked up and down with it in their shoe and we know it works in their shoe as well. Is there any merit in that?

P4: But if you already made, if you already made the device, then what's the point in asking them to make?

E.C.: Because it looks like like a dog chewed it. Like it's just not the finish. It's just not got a nice finish to it and it's not robust, it's not durable, it's sort of multiple layers. Surely the features, the features of that device are going to be so different to what they make, if it's going to be, if it's made out of different materials and different density. So what you're making is sending off there might act completely differently to what they're going to send you. But that's my point. You see if I've if I've gone for going. With XXX model of um, soft, medium and hard or low, medium and high and I've chosen a soft material or a medium material or a hard material, I should be able to emulate that in in a prescription with those sort of descriptors. It's just a thought and I'm just thinking in terms of building confidence in in the younger podiatrist who I’m sorry no P3 you go

P3: sorry I was going to say that sounds like very much like the technological version of what we used to have, when I first qualified in that we had our own lab and you'd make, um, some insults for patient. And back in the day we could all rotate in sort of half a day every couple of weeks to make um, some insults. And that's how I learnt what worked, what didn't work, what adjustments needed to be made and things like that. And to me that was an absolutely invaluable part of my, you know, early career. And I think that is what is missing today is because we have to, we don't have an option, we don't have our own labs. We have to send it to somewhere else. And you don't know that it's not. As I said earlier in the conversation, you don't know that it's not right until you get it back and the patient doesn't get on with it, whereas you with your scanning stuff is kind of the the posh version of what we used to do.

E.C.: Yeah, so I'm guessing. Sorry, sorry, p5.

P5: I'm guessing that would be the equivalent to our cardboard template then P3. So we make it in power on, we draw it on cardboard. We draw the positions that we want, the things. We draw where we want the bevelling. Yeah. And then we send it off. We want this made with this material in this place. With this cover and this base, it's close to that.

E.C.: The difference is that I'm proposing here just as a concept is that you actually build something that the patient has because they work. They, they, they've still got a piece of cardboard in their shoe at that stage, haven't they? Just got drawings on it. That's all I'm proposing that we actually dummy up a sort of a fake version of what they're going to get in the more you know. Durable, high quality, finish version.

P2: So why so? Because when I went to university it was we were like we saw the patient, if we want to fill those with, cast them, we then go to the lab on uni, make them ourselves, and then we got to see the whole process, and I'm a big believer in that. But that's now a very dated process because we've got cadcam 3D printed here. 3D print is only going to get more popular and I like the concept of having a beta test that we used to do it in southern health with Slim Flex. And then we then send off to the lab, but then we'd have the issues with the device we got back from the lab wasn't what I asked for. So we used to give them like four or five pairs of slim Flex just to keep them going because that was actually better for them because it just worked and it was comfortable That your concept demo and the cost of printing and materials will come down is why couldn't you be tested because 3D print something or mill something, just just don't finish it off, test them. OK, this is what we're going to do. It looks rough and ready, but at least it's the same material that they are going to have long term. And you get around the cost because that just becomes your orthosis package price. There's almost like when you go and get a tattoo done, you get the temporary one done first. You don't pay actually, it's just that's the cost of disservice, you see. Do you like it? Yes, I like it. Great. Now let's make it permanent. Similar concept with the filter faces, but let's use the materials.

E.C.: And you've known me too long. Well yeah, but also your experience is, is speaking. So yeah Jonathan you've got, you've

P4: got your I think yeah I I sometimes again probably did this more so when I first started working MSK it was the using prefabricated device first because I wasn't so confident in my custom made device sort of prescriptions and manufacturing those. And so yeah, I would do a similar sort of thing to what you're saying in terms of just doing adding little bits and pieces onto prefabricated device first. Four, then requesting a custom device. But rather than scanning the device itself again, I still would go through the process that we've talked about already in terms of scanning the foot and then attempting to with language to communicate exactly what I want to make from that. So now I think about it. I mean, again, what would be even better is if we could just go straight for the well again. But then maybe it wouldn't be because if you did make a custom made device, straight off you print it, then the clinic, fantastic, brilliant. And then actually, yeah, they didn't like it. They say then your process might have been better to have a sort of tester.

E.C.: On 1st. So I suppose how events, how, how confident you are on it or you know. Yeah, I suppose, yeah, it would be difficult to make that judgment. I don't know how you'd put a hard and fast rule that there probably isn't. That's probably the truth, uh. But yeah, so the reason I brought it in is into the conversation is because there's a lot of podiatrists, especially in the XXX, less so in XXX and I imagine less so in XXX as well from what you've said, XXX. But in the UK there is a lot of avoidance. Of MSK, it's almost seen as optional in practice and yet it is a core part of our, of our role and to be confident in it as well as competent, I think is where I'm heading with this conversation. How do we build confidence and competence so that they get to your stage, XXX, where they're just like, oh, I just know it works now, I've been doing this so long. I know my system, I know my materials, I know what works and my patients. There's been a process that you've been through to get to that. You didn't start there and I worked with undergrads. No, they don't start there. So I guess I'm asking you, you know, what is it that would help to build that confidence and competence? I I think it's. Sorry. Just let XXX come back in, Jenny and we'll come to you

P1: in a SEC. Sorry, sorry, P5, I I I think that you know, you you can't separate MSK from Podiatry to me. You know, everything is MSK, you know everything. You know and and I think that I mean I at XXX University in 2003, we actually signed a petition that we sent to the head of the department because we were heading towards the final months of our course and they hadn't yet started to teach us MSK. They didn't know anything about it. They didn't want to. And I'm going how can you Understand, um, foot pathologies if you do not know the anatomy and how the functional anatomy of the foot. And to me, I with every student I've seen on placement with every single one of the great band fives or I don't know what they are, but the younger podiatrists that I'm seeing with I'm going it is MSK. It's all MSK. It's nothing. It is it's MSK. Weight bearing is what separates feet from hands from every other portion. You can't. You have to be. If you have someone with a corn or callus or an ingrown toenail, you should be working out why and what's the musculoskeletal basis behind that pathology, even if it is a corn or a. Callus or a blister or some other thing, you know what I mean? And I think that to me, you know, I've seen now a lot of students on placement here at Southampton and honestly, I've, I've also had physiotherapy students. And. I know if you're involved in their course, I'm sorry if I if this sounds a little thing. But I mean I've asked them about their anatomy and they haven't got a clue. Yeah, you can do a students and I'm saying, OK, show me where your perennials are and they look at me with a blank expression. I've asked physiotherapy students a year too, and they know that stuff like that and I'm going, you know I'm and I really have been very surprised I have to say, at how weak these student that I've seen have been on anatomy. And to me, you start first with that and then work out corns and stuff. You know what I mean? If to me, I, I, I think, and certainly it was that way when I was trained, we had experts in diabetic wounds, none of it above all floating, none of it about them. Mechanics behind that wound. And we need to change that, in my opinion.

E.C.: Well, you're preaching to the choir with me. I totally agree with you. And I'm sure most people in this room do. Yeah. I

P5: mean, I think that's traditionally it's been postgrad stuff, hasn't it? It's been postgrad specializing, yeah. I would say that's probably where you refined your initial knowledge for sure, yeah. But also in the last few years, I mean, when I was at XXX, we went to the cadaver labs and that actually made a big difference because you could actually pick the leg up and move it and you could see the penny drop on people's faces as to what and how. And I know with COVID, you don't know their anatomy. Yeah, I know that you would, you would hope wouldn't. Yeah. But yeah.

E.C.: Sorry. OK well this is really helpful because I want to know what we can do to what we're talking about is modifying yes load but also function as well through that load where you know there will be you know part of the some of the purposes that we'll be using our for authorities for is to modify function as well. So that is sort of adapting functional anatomy on you cannot. I I cannot agree more with what you're saying about how we do that. And this is actually a living, breathing way of doing that. They can actually begin to envision the anatomy through trying to manipulate it. And I think that is probably integral to how we. Start to think about teaching anatomy, maybe in the future in Podiatry. So we are now at 6:00 o'clock bang on, and you've given me some fantastic pearls of wisdom. If you're desperate to get off the call, please do. Don't feel you have to stay. But if you do have a few minutes more to spare, I'd just like to hear final words, final thoughts, things that you're desperate to get off your chest that may or may not be anything to do with the questions we've asked, just anything to do with this

P2: And for me, it's it's an area that as podiatrists with bias, because we're passionate about it, because it is. It's the area where our training is in and foot. Orthoses have such a bad Rep within the medical world and within patient world as well. But I think a lot of the time it's not the device, it's the clinician, worry, podiatrist, osteopath, chiropractor, the title profession doesn't really matter. I think that we need to be better educated on why. Always asking why are we giving this and what we want to achieve by giving someone thought thesi then it's then working OK well see if you've done that. If you then know you're anatomy, if you want to offload the perennials then you can work out OK do I use a lateral scar to use a lateral heel cup? Do I use a stiff lateral border of the flexible medial border? You got so many. As P4 said there's so many ways to skin Am cat when it comes to that, but I think it's working OK how do we then make that any to repeatable reliable way and I think this is where 3D printing can be helpful. What I I completely agree with P1 that designing your own is going to be the way forward. But I think even one step further than that, I think actually once we get enough data and you start using AI to then start looking at prescription habits and looking things like, you can then start predicting what people are going to need by using technology. But to do that you need data and you need a solid foundation to start with. You can't just build that first. You need to do these baby steps first. I will take part of that.

E.C.: Point on board P2 but I I'm

P2: Going to be contentious and say that I think P3's other point about Neuro neuroanatomy basically how the patient is going to respond and their whole paradigm about therapy is going to impact that knowledge as well. So I think so I think we can build that into AI once we start looking at looking at patterns at previous history because that's the one reason you don't get with the labs. You don't get people's preconceptions you don't know that they've read 7000 Facebook posts and. Members of I hate orthosis groups on it and it's they don't see the patient that comes in with a suitcase full of previous yeah foot orthoses and it is that you've got to remember your trees in the human being as well as the foot and that's the bit overruled that I think not just in football authorities, I think in in Podiatry and healthcare as a general we don't do, but actually I think we have the capability to be better and capture more data on that.

E.C.: Thank you so much. But I know P1 you were alluding to that right at the very beginning where you're saying it's something I can't even begin to put into words by just no. And you've also alluded to it P3 with the patient response which is somewhat predictable and sometimes just really is not. So again, any final thoughts from you guys just before I come back to P5 and P3? Great.

P1: I I I think that certainly um with with what the way I work is that equally as important is like a a follow up appointment and and and you know in many many cases we make small adjustments to that based on and you know that is how we fine tune these devices the we don't just have to make something for the foot because the other thing is we have no control over the shoe they're going to put it into. The other thing I've also just want to very briefly touch on is that. The the actual exterior surface of the orthotic, as P3 said earlier about this thing, looks like a canal boat, but it goes further than that. It goes on the shape of. Around the heel Cup I would use a different back Skype for different pathologies and no lab. I've never found a prescription form that has how am. How would you like us to back Skype that heel? And then for what shoes? So I've I've a marathon runner, I've made thousands of orthotics for runners, and I know that every different brand has a different interior contour and the orthotics going to sit differently. In that way it is a very difficult. Bob and I think that by I just want to get back to the original thing. You know we have to hang on to that link between the the device and us you know we are the experts here and it's being taken away from us, you know every single. Put this practitioner out there that is making selling time, looks at it at an orthotic device as their lunch and they go, oh, I'm going to sell these things from make a fortune. But we are the experts in that and since coming back to XXX after 20 years away. I I see we're losing it, you know, was that the, the, the, the science and art of prescription design manufacture of of bespoke functional orthoses is slipping out of our hands. And it will be. I think we're giving it. There are some there's a generation that just wants to tick the box and we are we are giving it away and you know we're going to find ourselves and it will be our own fault and I think that the way I would start this out is getting there early. You know put biomechanics back into the front end of a university course enabling these kids are good at at computers you know get them onto CAD Cam design early. I will be more than happy to come to XXX University and show you and even give a demo. To the to your students. But, you know, get back control, take back control and brilliant way to end. I I love it. It's our, it's our property, it's our expertise and it is our area of expertise and we need to hang on to it like a cornered Pitbull.

E.C.: Right. That's what we are cornered pitbulls. I'm loving it. How about you, P4?

P4: Yeah, I mean, again, I probably get a lot of repetition here, but uh, yeah, I think that, yeah, you can't be prescribing a device until, yeah, you can take a good history. You understand all the sort of systemic conditions they might have and not how that might affect them. And also, you know the, you know, all the other aspects of their expectations and beliefs and then, you know, absolutely, yeah, anatomy, cadaver labs, you know, doing your. Sort of uh, Boney landmarks and drawing on people's feet and getting a really, you know, good grasp with that. Until you've got all of that, I don't think, yeah, you can. You can confidently make a device and have an idea of what you're trying to achieve and what's going on, and I think it's just putting it down to chance. And then, yeah, I think that as well. I think that something that uh I think that sometimes what you know I think the I think there was a transition from saying you know calling everything sort of biomechanics and moving towards MSK and my understanding of that was that absolutely by mechanics is an important component of managing patients with MSK pathology. But it's certainly not the be all and end all, it's not the same. It's only one aspect of what we do is we do so much more than just that and it's again in itself is a huge area but you know we do so many other you know sort of modalities. And treatments and you know that I think that you know that's that needs to be recognized I think as well when you talk about footwear, I think yeah that's probably the thing that's changed again most from when I started working MSK is that I put you know footwear for me is so, so, so vital. I think you know you have the best insult in the world, you know if there is a best one for that person but if they're putting it into a really flimsy slip on little shoe, it's just it's probably not going to make much of a difference. And so I think the marriage between the right kind of footwear actually I think that's again something where that brings. Whole another layer of complexity is to you've got a device you think works well for them and like our stuff, but then how does that interact with the shoe? And then often even when you get 3D gait analysis, I know that P2 works with some of that sort of stuff. That, uh, you know, even with that, again, quite often you're just looking at shoe deformation rather than the foot, foot position and posture and function as well. And so there it seems so simple, but whenever you dig into it, there's always another layer of complexity. And so I think that's what makes it fascinating. That's probably, possibly why we find this subject so interesting and really enjoy what we do. But yeah, you can be incredibly reductive and just say, OK, we're just going to get someone to walk onto some paper with their wet foot and see what impression that. He's not then dictates exactly what type of insole we're going to give to them with no consideration to anything else. But people still do that. One of my senior physiotherapist colleagues actually said that to me the other day that he did that. And I was like, what are you doing? Are you not listening to me over the past like million years? And so, you know, I think that all this stuff these come into and I think in terms of, you know, looking forward for students, I think, yeah, definitely, you know, making MSK a pillar of that, of their education, like you say, relating that tissue viability, relating that to surgery. And once someone had an operation how does that affect the you know the foot and the individual and how they move and and work. And I think that yeah there's a lot more to be done there and I think I completely agree. I think it would be really valuable for me to have had play playing more with CAD Cam machines. It's not something that I've had exposure to and I really would like that and would like to do do more of that and I think you know that would benefit students hugely and that that's pretty much it really won't go on anyway.

E.C.: That's fantastic. I love your energy, by the way. OK. Uh, P3 and P5, feeding off what the guys have said so far, do you have any of your own thoughts aside of that? Do you want to build on it? I'm, I'm particularly interested as well in what you were talking about Jenny, in terms of the the poor and the comfort side of things as well. So bring me what your final thoughts are?

P5: Yeah, I mean I. With the XXX and stuff, it's a case of mainly elderly people. Um, you know they've got away. They've got false knees, false hips that, you know their joint and range of motion and, you know, centre of gravity. All of that's changed. Put functions changed as a result. So I think there could be a lot more sort of education. I mean, even about things about centre of gravity. So if the heels aren't hitting the floor, they're going to be walking on the front of their feet. Do you know what I mean? So the. My experience at Uni was AP was just taught dry, it wasn't functional. And every time I tried asking questions it was like. Let me ask you that question for that's not that's you in your previous life. That's not you. As a podiatrist, you've got to forget everything you already know. And this is the way you've got to do it now, so. There needs to be more more, not just understanding of how normal body would function, but being podiatrists we're gonna be dealing with abnormal body function.

E.C.: That's that's a very interesting point. I outlined our anatomy teaching spectrum the other day. Believe me I obsess about this as much as you guys do and um you know working from that point of gross anatomy down to cellular anatomy and function to understand healing processes and that kind of thing. But right the way through from gross through to functional and then functional into pathological functional anatomy and that is a knowledge of anatomy that is close to radiological anatomy which is obviously where. If you're going into imaging, you need to be, you know, have a totally different understanding of pathological anatomy through the lens of a different imaging modality. So yeah, I couldn't agree more, Jenny. And that's really helpful to hear again, how important it is to integrate knowledge of anatomy with the clinical practice that we are trying to manipulate, manipulate the anatomy with our clinical practice. Is that you pretty much are there. Can I move to P3?

P3: Yeah. I mean, I think everybody's pretty much said everything that I was going to say because it. From objective it's teaching the the new graduates and the students really the fundamental importance of what is normal. Therefore you can understand what's abnormal, but it's not just looking at the foot, it's looking at the whole lower limb and body because you know the way people. Walk affects every part of them and whilst we focused in on the foot problem we need to understand the interaction of the the rest of the body with that foot because that will affect the function of the orthotic. The footwear has been said, you can have two seemingly similar issues on the surface but there will affect the orthotic in different ways and therefore affect its functionality and from the student you know perspective I think. Since. This is very sweeping statement and it's not meant to be overly critical, but I think since it's become a modular degree in that you do your diabetes, you do your anatomy, you do your whatever time and time again we get students that come on placement, not just from XXX because we previously had them from XXX that you would put them into bar mechanics or MSK clinic and they go Oh no, I don't need to do that. I've done that bit of the module. I don't. I don't need to learn anymore about that. And they don't seem to tie in the importance of functional anatomy across the spectrum as, as you know, Chris said with, you know, callous or corn or whatever, you know, you need to know the functional anatomy to know what you're doing to try and treat it. Otherwise you just turn into a cut and come again, thank you very much. Go away and we'll see You in six weeks and not again. Something that's dear to my heart is people are scared by MSK and biomechanics because they see it all as I must cast. Foot I must do a you know a fully supportive device they but they don't understand why they're doing it and equally I mean I I give equal numbers of non casted insoles to casted insoles because it depends on the pathology I'm treating and that individual and their footwear and everything else exactly right.

E.C: So yeah I think we're arriving at a consensus here that some certainly integrated learning is required linking the. Um, practice of foot orthosis, prescription design and production and then fitting and evaluation of it to the anatomy you're trying to manipulate, but with the overlay of complexity of being human and the sensations of pain and experience and expectation and all of that plus. The influence of footwear and activity, um, even the effect of tiredness and how you might run at the beginning of a race and at the end of the race, which is what I'm interested in, um, all of that kind of stuff is complexity upon complexity upon complexity. And so for that reason I'll tell you where I'm at just to sort of show my cards and I hope this will resonate with you. I firmly believe that we need to take a case based approach to learning right from the beginning we set out with simple cases with Simple problems to solve and we just complexify and complexify and complexify. So they get used to building that same foundation in but then bolting more on bolting, comorbidity on bolting age on bolting expectation and yellow flags and health anxieties on and whatever it is So maybe that is a a learning package that can can begin to come from this project as well. Oh my gosh guys, I want to take you home with me and give you a glass of wine because you've just been so phenomenal tonight. Thank you so much for your time, your wisdom. Expertise, your honesty and your vulnerability, um, in this process, um, we will definitely stay in touch. And um, hopefully, um, some good stuff will come out of this. And I and I will absolutely make sure that you see where you're impact landed with that. So go back to your lovely lives, enjoy your evenings, and I look forward to seeing you again soon. And thank you.

All: Bye, bye, bye, bye, bye, bye, bye.

**Supplementary file 4:** Six Phase Guide for Thematic Analysis

Step 1: Become familiar with the data.

Step 2: Generate initial codes.

Step 3: Search for themes.

Step 4: Review themes.

Step 5: Define themes.

Step 6: Write up.

Braun V. and Clarke V. (2006) 'Using thematic analysis in psychology', *Qualitative Research in Psychology*, 3(2), pp. 77-101.

**Supplementary file 5: Coding process**

*Patient Focus Group*

Overarching themes:

Mixed experiences

- P2: “Got one set, had some analysis done put my foot in the sand got measured up but weren’t very good at all. Arthritis got worse so I got another set which were much better. 2 different experiences, the 1^st^ set short, firm plastic and hard. Trauma from them reverberated through the body, I even got a retinal tear in year. Could just feel how hard they were and vibrated through the whole body. Now I have something that is softer full length and much more supportive and feels a lot more comfortable. So, 2 different experiences. hasn’t completely solved the problem but getting there.”
- P2: “I went to the first place for analysis and practitioner said you would benefit from some of these but like I said tried them hoping being optimistic but carried on but it weas bad experience. Then I went to have some injections across the joint but didn’t seem to make a difference. Consultant said come here as might get different options, that was better experience.”
- P3: **“**I used to wake up in the morning dreading putting my feet on the floor… these lovely leather ¾ length to put in shoes. Within 2 weeks my feet were sound it was like this was heaven for me… They went strong for a very long time…I wore them for everything.”

Process

- P2: “Maybe there is a better solution like contact lenses instead of glasses not an issue for me just something you have to do.”
- P2: “The whole thing especially with NHS you have dental check 6 and eyes retested I think even more important with feet as more important part of body but what you get in orthotic cam be changed overtime you change so even more so than your sight or teeth.”

Technologies

- P2: “I wanted to see the mechanics, see the skeleton foot I wanted to see the whole joint, but I couldn’t really get it. So similar experience though here more technical and I quite like that.”
- P1: “When go to podiatrist I found most had tiny little room with treadmill and told by 1 that you can’t see how anyone walks with the ground underneath you. You have to go over the ground to walk properly.”

Roles

- P1: “You see them as professional, but I feel like apologising when not working properly. You have to put up with a bit of discomfort and you think it is your fault.”
- P3: “I know best for me.”
- P2: **“**They are the expert, you come, they give you something and say on you go but you might get chance to come back.”

Involvement

- P2: “It’s important to have that voice and give that feedback because things change all the time.”
- P1: “Yes it was, I felt my feeling couldn’t get over to anyone… I think I keep saying things. Right foot is toe dropped down kept saying need support on that side but couldn’t get across to anyone.”
- P2: “I would have been good to have more time. You have your 15 or 30 min appt for fitting orthoses if fit ok you’re done. I would like to have had 5 mins on treadmill or test foot orthoses with sport specific movement to see if had a difference at the start because if I put squash shoes on and run lunatic might have discovered too high, too firm, too slippy for movement not straight forward and I’m going over my ankle”
- P3: “If you have an orthotic and you think you need something. You think you need a dome thing or seething added or taken away quite easy with stick on stuff but if you’ve got something that’s bespoke and focussed on your particular foot you kind of feel should have time and space to say this is my orthotic it is giving me jip on this side is there something we can do to it there and then and have that time.”
- P2: “I think to have more time to talk. It is quite focussed you’re in there, get measured, get stuff next time is it all ok yes then you’re done., it’s quite clinical and targeted. A little more stepping back, discussion like what’s your experience, what’s it like when walking, standing support might just draw out things that shapes what you get.”

Cost

- P2: “Yes, **X (the podiatrist)** said get a 2^nd^ pair, get £30 off.”
- P3: “Financially not afford it”

Transferability

- P2: “For me difficulty is you only have 1 set so have to keep taking them out. Went for run on Tuesday and it rained and rained so they are so got so wet so you have to walk around in them either in wet shoes or don’t have them and feet get sore so what do you do? Good to have efficient way to have more than 1 pair even if same pair printed but for me.”
- P1: “It would be good to have 1 printed for every shoe.”
- P1: “Gave me a slimmer pair for nice shoes but I can’t have nice shoes as front of foot too wide. They don’t do smart shoes for wide feet. The whole foot isn’t wide it’s just the forefoot which is wide. My ankle is slim.”
- P2: “Different pair for different shoe I went yeah squash shoe lower, running shoes firmer walking around. It could be a different pair for each shoe but definitely better to have more than one. I don’t think there is any going back feels much better with them.”
- P2: “Ultimately, I want to scan the thing [foot] at home and have lots of pairs for walking, work, sport.”

*Podiatrist Focus Group*

Overarching themes:

Confidence

- In the process:
  - P1: I have absolutely no faith in this process in that the what I prescribe is not coming back and it doesn't it really many of the orthotics. And they come back, have to be redone or refitted.
  - P1: On the other hand, I have a private practice where I have my own orthotics laboratory which I have 3D machines and there I have complete confidence that I know the person is going to get what I meant, what they need.
  - P3: When it works, it works well. I mean, we we're fortunate, I suppose most of the time we get our devices back within four weeks
- In the lab
  - P2: what's the point to me writing a number he says I'm just going to write small medium and large and it's not ended up doing
  - P2: working in any field, you're gonna have to have a certain amount of confidence and obviously sometimes misplaced. But at least I want to know that I'm going to get what I've ordered.
  - P4: that it does take a little adjustment period to to, yeah, get used to what their prescriptions like saying like the, say, the terminology, which is different between different manufacturers.. you get to a point where you do feel a little bit more comfortable and you feel like you have a better idea of when you, you know, send off that device, what's going to come back. And you get fewer surprises with that
  - P4: my confidence grows with a particular manufacturer as you've worked with them for a little bit longer
  - P4: certain labs as well that they're really good with. And shell materials and then others they're not so great.
  - P2: moment I like that relationship that you've spent time building because I have had arguments where I have phoned owners of labs up and said look, this is a pile of crap you just sent me. Get your ideas up and I want this sorted
  - P5: they would take thefoam box, they'd scan in the impression, and then they would flatten out all the lumps and bumps. And I'm like, but what? What is the point? And they're like, no. If you want the lumps and bumps, you have to do a cast. OK. And to me that just seemed like a. Massive missed opportunity.
- In yourself
  - P1: you have more confidence in yourself than the lab you're using
  - P4: You can confidently make a device and have an idea of what you're trying to achieve and what's going on, and I think it's just putting it down to chance
- In students
- Clinician preferences
  - P2: I I still, I do believe that we should be the ones designing. We're the one who's got the foot, the patient in front of us, we're the ones know exactly what we want.
- Clinician variability and manafacture’s perspective
  - P1: some of the casts that arrived, they don't even look like they've been taken from a foot.. he's like, how on Earth am I supposed to.. and try to design like read the podiatrist.
- Reputation of foot orthoses
  - P2: Orthoses have such a bad Rep within the medical world and within patient world as well.
  - P2: You don't get people's preconceptions you don't know that they've they've read 7000 Facebook posts and. Members of I hate orthosis groups on it and it's they don't see the patient that comes in with a suitcase full of previous yeah foot orthoses and it is that you've got to remember your trees in the human being as well as the foot and that's the bit overruled that I think not just in football authorities, I think in in Podiatry and healthcare as a general we don't do, but actually I think we have the capability to be better and capture more data on that.
- Building Trust:
  - P2: it's imperative that you build up trust in the lab so that you have confidence or at least more confidence in them
  - P2: us I would say the amount of modifications we have to do are very minimal because I think it goes back to building that relationships with. With the labs.
  - P1: we have to hang on to that link between the the device and us you know we are the experts here and it's being taken away from us

Process

- Learnt
  - P4: it's a learning process
  - P4: I generally enjoy the most of the process and then yeah, like I say, when it obviously when it goes well, it's it's really, really great.
- Adapting
- Taught
- Belief
- Challenges/obstacles
  - P3: some of them were coming back like canal barges… huge, huge problem because most of them were going back to be remade
  - P4: [3D Scanning] a little bit more of a challenge because you know usually when you do the impression box it's either you know full weight bearing or something weight bearing to a certain extent even if you're pushing it down with your hand. Whereas yeah you can do it completely non weight bearing with a plaster of Paris or 3D scanner you can manipulate the foot as well, slightly as well. And so I think that then I have to make an adjustment with my prescription as a result of that depending on what I'm trying to achieve.
  - P4: , it's still always there those challenges there and I think I get most frustrated. Yeah something does come back that isn't what quite what I had imagined or that you know the patient has responded differently to how I'd imagine and again that was. That's got a learning point for me
  - P1: then looking at the prescription sheet design on CAD, the computer aided design, they then design the orthotic. They then interpret what you've written on the prescription form coupled with the foot impression that they have and they make the authority correct and what, how, how are they getting it so wrong?
- Prescription forms:
  - P1: Almost any prescription form is that you're essentially giving away your intellectual property to some person on the other end who you lose that connection.
  - P1: despite the loads and loads of orthotics labs and lots of work that's been done in trying to design the perfect prescription form, I don't think that exists.. they're too complicated. It's almost like they want to take the job away
  - P1: And I mean, I think the the current form we use says it says the PMP and they there's no way you're going to put it… Something like a PMP is an incredibly subtle and powerful tool that can be make a massive difference or just be a painful lump under your foot. Exactly. And it's critical that the placement of the shape that the the thickness, these things are.
  - P4: I don't think there is a perfect prescription for any individual
  - P5: The forms are quite good because they do try and make you think and they kind of give you a tick list to go
  - P1: [CADCAM] It is unbelievably simple, and I cannot imagine filling out a form privately ever again.
- Extremes
- Control
  - You're making it exactly how you want it. You can you know, do the CAD Cam adjustments or yourself and that's it. And like you say, you're hopefully, you know, mitigating. Some of the risk in your, uh, controlling some of the variables like I say, of going through the communication through other people and all those other things. And you've got a lot more sort of control over that
- Follow up
  - equally as important is like a a follow up appointment and and and you know in many many cases we make small adjustments to that based on and you know that is how we fine tune these devices the we don't just have to make something for the foot because the other thing is we have no control over the shoe they're going to put it into.
- Variation in footwear
  - P2: I've never found a prescription form that has how am. How would you like us to back Skype that heel? And then for what shoes? So I've I've a marathon runner, I've made thousands of orthotics for runners, and I know that every different brand has a different interior contour and the orthotics going to sit differently.
  - P4: footwear for me is so, so, so vital. I think you know you have the best insult in the world, you know if there is a best one for that person but if they're putting it into a really flimsy slip on little shoe, it's just it's probably not going to make much of a difference.. I think the marriage between the right kind of footwear actually I think that's again something where that bring..soften even when you get 3D gait analysis, I know that Nick works with some of that sort of stuff. That, uh, you know, even with that, again, quite often you're just looking at shoe deformation rather than the foot, foot position and posture and function as well. And so there it seems so simple, but whenever you dig into it, there's always another layer of complexity. And so I think that's what makes it fascinating

Differences between NHS and Private Practice and other disciplines e.g. orthotists/opticians and other countries

- Time receiving orthotic:
  - P3: a lot of these labs were set up with orthotists in mind, not podiatrists.
  - P1: I've always been very jealous of opticians.. it's so easy the computer tells them the the prescription, the machine makes it and it comes back three days later and generally they work and we are dealing with
- Taking impressions cast/foam/3d
- Funding:
  - P3: We've changed labs say three times and that has been out of our control because the way Dorset Healthcare works, we are working with some of the orthotics departments as well. And so the contract was put out to cover both aspects of it. And so contracts were changed for nefarious things like they wouldn't deliver at a certain time to a certain place.
  - P5: I mean I work in a different part of Dorset to Nikki and we don't have access to the same things… If I can't make it out of poron. It doesn't happen
- Technological advances
  - P1: So when I got involved in CAD Cam technology but eight years ago, So what I got was the whole process, the machine, the software, the So what I've. I designed the orthotic myself. You know, it doesn't go to the lab.. you can shape it, you can cut out, you can look, you look at it and go. Now that's what I want in the position I want for that.. There's no argument about whose fault it was if it didn't work because they designed it. And for me, I'm I my way of making orthotics is that I will design it Millet in the machines I have, and the patient gets it four days later. I can't imagine doing it any other way, quite honestly.
  - P3: it it is great being able to work on the machine and and design it you know as as Chris does because what you see is what you get and having our own lab made life so much easier because if if things didn't work you could get the alterations done quickly
  - P4: I'm trying to shift everything over to doing it digitally… in private practice the 3D scans, but bring it into the NHS as well. And I find that that does adjust my prescriptions.
  - P4: it gets a quicker turn around more environmentally friendly and also you getting a very accurate impression of the foot as well
  - P2: . The digital scanning again I've been, I've been doing it for six years now using the structure sense scan again that makes my life a lot easier
  - P4: hopefully not in the too distant future where you take the 3D scans there and then in the clinic you then have your own 3D printer and it just prints out there there for you. And then I don't know how long it would take, but then you could just have them all done there like say all in-house
- Pricing

Communication

- Language:
  - P1: the vocabulary is insufficient
  - P2: All the language is different… everyone's 5 degrees is slightly different.
  - P2: sometimes I don't even use their lab zone form. I write it in my own language and they know what I mean. Which is OK for me, but that's not scalable at all for a profession because only I've got my brain. No one else has access.
  - P3: But once the lab gets to know your language you do get much more consistency of devices, but you you get variations amongst clinicians and and what I ask for and what my colleague asked for although we think we're asking for the same thing, if we use slightly different terminology you'll get different things back. So there's a lot of factors you know that affect the orthotics and how we get them when they get them
  - P2: The universal language of what does a PMP pad look like? What does a first met cut out look like? So actually, if you ask of something. You get a standardized item back.
  - P4: Only a consistent language. Across the board would be really, really useful. And I think in an ideal world, I mean I think this will probably will become viable at some point.
  - P3: we are sending, you know, although we do very similar things, we are sending things with our language. And the labs are used to working with the Orthotists language, which you can say the same thing, but it means two different things.. . If they've had years of experience of working with orthotists, they are tuned into to what their interpretation of that particular terminology is, which is different to ours. So I think if you know if we could change one thing that if we're going to continue with external labs, if we could have these labs run by podiatrists for podiatrists because then we would all speak pretty much the same language with
  - P4: I still would go through the process that we've talked about already in terms of scanning the foot and then attempting to with language to communicate exactly what I want to make from that.
- Disconnect:
  - P1: I think there's a real communication breakdown.
  - P2: the biggest issue we have is to breakdown the communication.. because we're trying to write down on a bit of paper this 3D living bit of tissue and say I want you now to do this for this person that you're never going to meet. You're never gonna speak to you. You're never gonna know how they are, how they react to things or anything. And I want you to design this. For me and this sort of like you would never. You would never work in any other industry like that
- Misinterpretation:
  - P3: A lot is down to the lab interpretation.
- Frustration
  - P1: there's a little bit of extra that I just know that person needs that I can never communicate. To a lab, no matter how hard they try.
  - P3: it it's frustrating me and it's making it very difficult for the less experienced podiatrists to know how to have confidence in their own prescribing because they're they're getting things back and
- Building relationships
  - P1: it's incredibly primitive. It's complete potluck as to what you get back.
  - P2: it's that communication. So This is why I'm I only work with set number of labs and I don't care another lab would come back and sell me a product for half the price. I won't move because that lab knows how I communicate.
  - P4: communication with the lab is paramount.

Affects on students and newly-qualified Podiatrists

- Teaching at University
  - P1: . I'm not seeing a lot of podiatrists that are sitting down doing the CAD. And I, if I could change something, I would say teach it in university.
  - P4: CAD Cam designer at university would be really valuable.. much greater sort of depth of understanding of how these devices are made and and again you know how how what, how the device might return and what they might be thinking about with their prescriptions at that level
  - P3: And I think that is what is missing today is because we have to, we don't have an option, we don't have our own labs.
  - P2: university it was we were like we saw the patient, if we want to fill those with, cast them, we then go to the lab on uni, make them ourselves, and then we got to see the whole process, and I'm a big believer in that.. that's now a very dated process because we've got cadcam 3D printed here. 3D print is only going to get more popular and I like the concept of having a beta test that we used to do it in southern health with Slim Flex
- ‘core podiatry’
  - P1: how can you Understand, um, foot pathologies if you do not know the anatomy and how the functional anatomy of the foot… If you have someone with a corn or callus or an ingrown toenail, you should be working out why and what's the musculoskeletal basis behind that pathology, even if it is a corn or a. Callus or a blister or some other thing, you know what I mean? for students, I think, yeah, definitely, you know, making MSK a pillar of that, of their education… I think it would be really valuable for me to have have had play playing more with CAD Cam machines. It's not something that I've had exposure to and I really would like that and would like to do do more of that and I think you know that would benefit students hugely and that that's pretty much it really won't go on anyway.
- Anatomy knowledge
  - P3: since it's become a modular degree in that you do your diabetes, you do your anatomy, you do your whatever time and time again we get students that come on placement, not just from Southampton because we previously had them from Plymouth that you would put them into bar mechanics or MSK clinic and they go Oh no, I don't need to do that. I've done that bit of the module. I don't. I don't need to learn anymore about that. And they don't seem to tie in the importance of functional anatomy across the spectrum as, as you know, Chris said with, you know, callous or corn or whatever, you know, you need to know the functional anatomy to know what you're doing to try and treat it.
- P3: It would knock the confidence of of some of the more recent yeah recently qualified podiatrist

Broad scope of orthotics

- Science and art:
  - P1: they always say biomechanics is a mixture of science and art. I think that you lose an awful lot of the art by letting someone else onto your turf
  - P2: there is definitely that blend you know, it's blend between art and and science
  - P4: the other sort of nuance probably where the art comes into to what we do is that we don't know for sure exactly how patients are going to respond to devices.
  - P4: The science and the data just, you know, just to analyze everything and throw it all together and say this is gonna be the perfect device. There's, there's, there's, there's a human aspect to it… it's just counting on our experience, understanding the principles of understanding of that individual from the time that we do have with them.. I think my understanding is that there is a sort of like say a therapeutic window and it's just depending on that individual, you know, that could be really, really like say easy win or it could be really, really tricky and some people you might never win. sometimes you have to just put your hands up and say you know that's that's just and that might be related to their expectations and their beliefs and their you know all sorts of different things.
  - P1: the science and art of prescription design manufacture of of bespoke functional orthoses is slipping out of our hands.
- Individual to patients
  - P4:In my NHS days. A patient coming for review and said, oh, the authorities are wonderful. They had the left one in the right shoe upside down, yeah, upside down. And they're like, these are wonderful. And I'm like, well, where do I go from here? Nice, yeah.
- Goals
  - P4: a big part of our job is enjoying it when hopefully when things go well and when someone comes back and they really, really happy with them and you know. You're, you're, you you've achieved their goals. And so yeah I I I enjoy the process of it and you know
  - P4: but I think that would give the best patient outcome as well in terms of consistency of device, quality of device and and then also there's return being so much more so quick.
- Theatre
  - P4: Patients like that experience as well because they think it's new technology when you tell them actually it's not that new and it just adds a bit more theater to that patient experience and then they come back and they see their foot and they're Oh my God, that's my foot…. It it it all becomes that nice journey for the patient
- Beta testing
  - P4: if you did make a custom made device, straight off you print it, then the clinic, fantastic, brilliant. And then actually, yeah, they didn't like it. They say then your process might have been better to have a sort of tester.
- Evaluation
- Future direction
  - P2: designing your own is going to be the way forward. But I think even one step further than that, I think actually once we get enough data and you start using AI to then start looking at prescription habits and looking things like, you can then start predicting what people are going to need by using technology. But to do that you need data and you need a solid foundation to start with
- MSK vs BMX
  - P4: I think there was a transition from saying you know calling everything sort of biomechanics and moving towards MSK and my understanding of that was that absolutely by mechanics is an important component of managing patients with MSK pathology. But it's certainly not the be all and end all, it's not the same.
